# Climate suitability for European ticks: assessing species distribution models against null models and projection under AR5 climate

**DOI:** 10.1186/s13071-015-1046-4

**Published:** 2015-08-28

**Authors:** Hefin Wyn Williams, Dónall Eoin Cross, Heather Louise Crump, Cornelis Jan Drost, Christopher James Thomas

**Affiliations:** Institute of Biological, Environmental and Rural Sciences, Aberystwyth University, Aberystwyth, Wales

**Keywords:** Species distribution model, Null modelling, Maxent, Mahalanobis distance, Tick, RCP, Climate change

## Abstract

**Background:**

There is increasing evidence that the geographic distribution of tick species is changing. Whilst correlative Species Distribution Models (SDMs) have been used to predict areas that are potentially suitable for ticks, models have often been assessed without due consideration for spatial patterns in the data that may inflate the influence of predictor variables on species distributions. This study used null models to rigorously evaluate the role of climate and the potential for climate change to affect future climate suitability for eight European tick species, including several important disease vectors.

**Methods:**

We undertook a comparative assessment of the performance of Maxent and Mahalanobis Distance SDMs based on observed data against those of null models based on null species distributions or null climate data. This enabled the identification of species whose distributions demonstrate a significant association with climate variables. Latest generation (AR5) climate projections were subsequently used to project future climate suitability under four Representative Concentration Pathways (RCPs).

**Results:**

Seven out of eight tick species exhibited strong climatic signals within their observed distributions. Future projections intimate varying degrees of northward shift in climate suitability for these tick species, with the greatest shifts forecasted under the most extreme RCPs. Despite the high performance measure obtained for the observed model of *Hyalomma lusitanicum*, it did not perform significantly better than null models; this may result from the effects of non-climatic factors on its distribution.

**Conclusions:**

By comparing observed SDMs with null models, our results allow confidence that we have identified climate signals in tick distributions that are not simply a consequence of spatial patterns in the data. Observed climate-driven SDMs for seven out of eight species performed significantly better than null models, demonstrating the vulnerability of these tick species to the effects of climate change in the future.

**Electronic supplementary material:**

The online version of this article (doi:10.1186/s13071-015-1046-4) contains supplementary material, which is available to authorized users.

## Background

Ticks are globally important as vectors of a wider range of pathogens than any other arthropod group, and tick-borne diseases impose a significant burden on human and animal health [1]. It is therefore imperative to identify the current distribution ranges of ticks, as well as their likely distribution in the future. This has frequently been addressed by the use of process-based population models and correlative species distribution models (SDMs) [2].

SDMs build a multidimensional description in ecological space of the conditions where a species may potentially occur, based on predictor variable values at locations where it has been observed [3]. This is projected into geographic space to produce a probability surface of suitability. The applicability of an SDM for capturing a species’ ecological niche is primarily assessed on its ability to predict the observed distribution; most prominent among methods used is the Area Under the Curve (AUC) statistic which is calculated from a Receiver Operating Characteristic curve [4]. The AUC rewards models for correctly identifying areas with observed presences as highly suitable and predicting low suitability where the species has not been recorded. Perfect predictive performance is denoted by an AUC value of 1, whereas a value of 0.5 would be expected by random chance [4]. A widely seen pattern in the SDM literature is the use of specific AUC values to indicate models that are considered to be ‘of use’; for example, AUC values of at least 0.7 are considered ‘fair’ [5]. However, SDMs are correlative models dealing with data that almost inevitably contain spatial patterns, such as positive spatial autocorrelation where values geographically closer together are more similar than expected by chance [6]. These patterns have been shown to inflate the AUC value that would be expected by chance from SDMs [7]. AUC values from SDMs have also been observed to systematically inflate when the size of the study area is increased relative to the extent of the geographical range of the organism in question [8]. This is likely to explain why SDMs of species with geographically restricted distributions generally attain higher AUC values and are adjudged ‘easier’ to model [9–11]. Consequently, SDMs with high AUC values and supposedly excellent predictive performance can be obtained irrespective of whether the models are identifying plausible or causal relationships between environmental predictors and the distribution of the species [12]. This heightens the risk of incorrectly projecting spurious correlations between species occurrence and environmental predictors to new spatial or temporal areas [3]. One method devised to combat this risk is the comparison of the predictive performance of the SDMs against those of appropriate null models that are identical to the model being tested, apart from the replacement of some observed data with generated null data. As spatial autocorrelation is frequently a feature of observed data, Beale and colleagues [13] and Chapman [14] have advocated that null data should possess similar spatial structuring to observed data. This ensures that models based only on observed data and models based on null and observed data are equally affected by spatial patterns in predictor and species presence data. Key in using resulting null models to determine the strength of associations identified by SDMs is the *comparative* assessment of the AUC from the SDM based on observed data against those from SDMs based on null data. In this case, the absolute value of the AUC derived from the model based on observed data, which varies depending on the species and study area [8–12], is not the primary concern. Several applications of null modelling techniques in recent years have been demonstrated to robustly assess the performance of SDMs [15, 16], but we are unaware of their use for assessing SDMs of disease vectors. Consequently, the application of this technique provides an important advancement in the robust assessment of SDMs constructed for tick species.

The selection of appropriate predictor variables is critical to the generation of robust and reliable SDMs [17]; it is therefore imperative to consider the ecology of the modelled species. Ticks are haematophagous and require one or more hosts to complete their life cycle, but considerable periods of their life are spent off-host (>95 % of life for most *Ixodes* ticks [18]), where environmental factors strongly influence their activity, demographic rates, and distribution [2, 19]. Key amongst these environmental determinants is climate, as discussed by several in-depth reviews [2, 18, 20, 21]. Although microclimate is a more direct influence on a tick than macroclimate, only data for the latter are available at this large spatial scale. Several authors have used macroclimatic temperature and rainfall variables to model tick distribution (*e.g*. [22, 23]), although it is also important to include a measure of water stress for ticks as this is not effectively represented by rainfall [24]. Saturation deficit quantifies the ‘drying power’ of the air [25], which can drive tick mortality through cuticular water loss [24, 26]. It has been suggested that saturation deficit better represents the constraining influence of water stress than relative humidity does (see [27]) and it has emerged as a better predictor than relative humidity in a recent population model for *I. ricinus* [28]. Ticks are known to seek favourable relative humidity in microclimates [18] where they may actively absorb water [29]; however, available data on macroclimatic humidity is not representative of that in a tick’s microclimate [30]. As ticks are ectothermal, temperature also affects development rates and activity [21, 31, 32], and temperature and water availability in concert can therefore influence geographic distributions. The northern distribution of *Ixodes ricinus* in Europe, for example, is thought to be limited by low temperatures, whilst high temperatures and saturation deficit govern its southern distributional limit (see [19]).

Whilst both process-based models and SDMs have used climate to predict tick distributions (*e.g.*[22, 28]), this study goes beyond previous work by testing the power of SDMs based on observed data against models constructed with null climate or presence data. We seek to ascertain if eight species of ixodid ticks in the western Palearctic demonstrate a stronger association with climate than expected by chance. These species are: *Dermacentor marginatus* (Sulzer, 1776), *Haemaphysalis punctata* Canestrini & Fanzago, 1878, *H. sulcata* Canestrini & Fanzago, 1878*, Hyalomma marginatum* Koch, 1844*, Hy. lusitanicum* Koch, 1844*, Ixodes ricinus* (Linnaeus, 1758)*, Rhipicephalus annulatus* (Say, 1821) and *R. bursa* Canestrini & Fanzago, 1878. These tick species are of medical and veterinary importance as disease vectors in the western Palearctic region, and a comprehensive dataset of presence locations has recently been compiled [33]. Although *D. reticulatus* (Fabricius, 1794) and *R. sanguineus* group ticks are included in this dataset, the former is excluded from modelling due to incomplete representation in the dataset of parts of its known range [33], and it is considered inappropriate to model the latter group as it is composed of species with substantially different ecological characteristics (see [34] and references therein). Following comparison with null models, those species that are found to exhibit a significant climate signal in their distribution will be modelled under the influence of future climates. We will investigate the potential effects of climate change on future climate suitability using four projections from the Intergovernmental Panel for Climate Change (IPCC) Fifth Assessment Report (AR5) [35].

## Methods

**Fig. 1:**
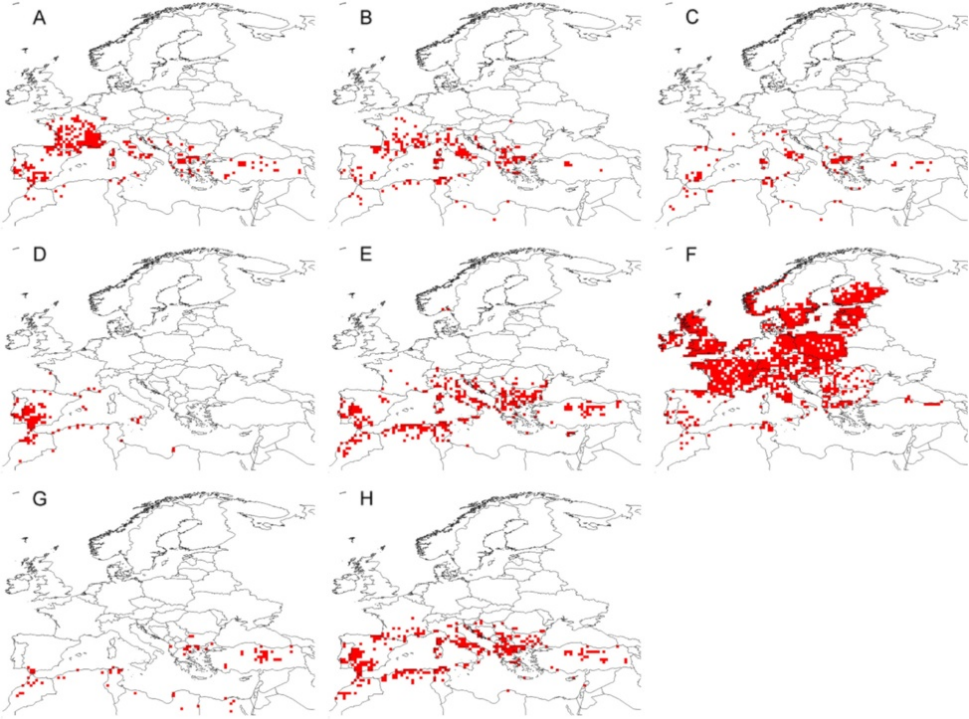
Tick species presence locations in the western Palearctic. Original data compiled by Estrada-Peña *et al.* 2013 [33]. Presence points converted to presences in 0.5 decimal degree cells. **a**: *Dermacentor marginatus*; **b**: *Haemaphysalis punctata*; **c**: *H. sulcata*; **d**: *Hyalomma lusitanicum*; **e**: *Hy. marginatum*; **f**: *Ixodes ricinus*; **g**: *Rhipicephalus annulatus* **h**: *R. bursa*

This study uses 8,501 geo-referenced records of eight tick species recorded between 1970 and 2010 which have been compiled by Estrada-Peña and colleagues [33] for the western Palearctic region as designated by those authors. Tick presences have been denoted within 0.5 decimal degree grids corresponding to the resolution of the climate data used in this study (Fig. 1).

The Climatic Research Unit (CRU) time series of high-resolution gridded data of monthly climate (Version 3.22; [36]) were obtained from the British Atmospheric Data Centre (badc.nerc.ac.uk). Nineteen core Bioclim climate variables (see worldclim.org/bioclim) were produced from the CRU data using methods described in Busby (1991) [37]. In addition, saturated vapour pressure was calculated from mean temperature using one of two Magnus equations [38], according to whether water was liquid or ice, with the assumption that dewpoint temperature was equal to minimum temperature for the crude estimation of wet bulb temperature (as per[39]). Actual vapour pressure from CRU was subsequently subtracted from saturated vapour pressure to obtain saturation deficit [40]. Average saturation deficit in spring and summer was used (‘summer’ defined as the warmest quarter of the year; ‘spring’ constitutes the preceding quarter), to reflect the restrictive influence of saturation deficit on tick populations during this period (*e.g.* [29, 41–43]. All 20 variables were averaged over a 40-year period (1971–2010) to correspond with the temporal period of the tick presence data.

The latest generation of future climate projections from four Global Circulation Models (GCMs; IPSL-CM5A-LR, MIROC-ESM-CHEM, GFDL-ESM2M, and NorESM1-M) have been prepared as inputs for the Inter-Sectoral Impact Model Intercomparison Project (ISI-MIP; [44]); these were obtained from the Earth System Grid Federation. The four Representative Concentration Pathways (RCPs) are named after the amount of global radiative forcing expected by the end of the 21st century on each pathway [45]. RCP 2.6 is a ‘peak and decline’ pathway; RCPs 4.5 and 6.0 represent ‘stabilisation without overshoot’, and RCP 8.5 signifies a continuing rise of CO_2_ (and consequently radiative forcing) beyond 2100 [45]_._ Data from each GCM for each RCP were used to derive averages of the 20 climate variables, following the procedure used for observed data, over the 40-year periods up to and including 2050 and 2098. All of the ISI-MIP variables used had previously been subjected to bias-correction [44], with the exception of relative humidity [46]. As it was deemed inappropriate to calculate saturation deficit from a combination of non-bias-corrected and bias-corrected data, non-bias-corrected mean and minimum temperature data were used to calculate saturated vapour pressure. Projected saturation deficit was then obtained by subtracting relative humidity from 100 % and multiplying this by saturated vapour pressure [40].

Climate variables from the observed CRU data were standardised (see *e.g.* [47]) before being subjected to a Principal Component Analysis (PCA) within the western Palearctic study area. This eliminated any multi-collinearity between individual predictors and reduced the number of predictor variables used in the SDMs, whilst retaining the majority of the variance contained in the climate data. Scores for the first three Principal Components (PCs) were used in the SDMs. PC scores for future climates were derived by standardising future data using means and standard deviations from standardisation of observed climate data, before subsequently applying the eigen vectors from the PCA of the observed data.

Many SDM procedures have been developed, each with its own strengths and weaknesses [48]. This study combined predictions from two SDMs in order to represent the consensus [49] across maximum entropy (Maxent) and Mahalanobis distance (MD) modelling approaches. Maxent is a machine-learning method that minimises the relative entropy between the probability densities of the species presence points and the wider landscape within the study area [50]. Its output represents the probability of presence of a species in each grid cell [51]. The internal fitting of Maxent models was evaluated to avoid overfitting, and the most parsimonious model was selected for each species (steps undertaken are detailed in Additional file 1). MD creates a multivariate mean based on the environmental conditions at the points where the species has been observed and gives a measure of dissimilarity at all other locations within the study area [52]. Raw MD output values were recoded following Clark et al. [53], and climate suitability of each grid cell was thereby assigned on a scale of 0 (low) to 1 (high).

**Fig. 2:**
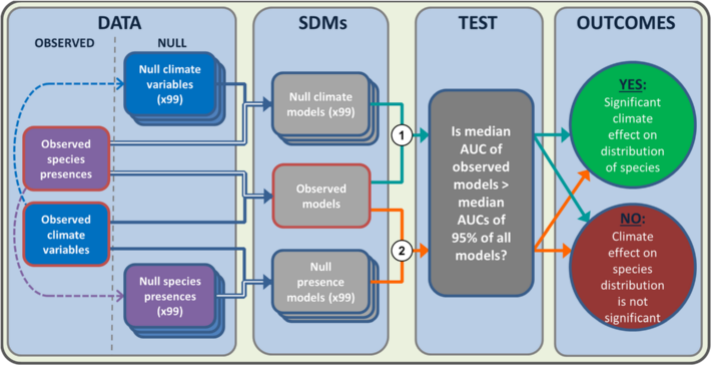
Workflow followed to construct SDMs and test for significance of climate signal. Null data were generated with similar spatial patterns to those present in observed data (dashed arrows; see Additional file 2). Observed and null presence data were split 1000 times into training and testing data (60:40 %; double line arrows) and used to construct observed and null models. Multiple post-hoc pairwise comparisons of median AUC values from observed and null models were undertaken using critical values from the t distribution to test for a significant climate signal. The workflow was followed once for Maxent and once for Mahalanobis Distance SDMs for each species, producing four independent outcomes per species; tests of observed Maxent model against null climate (1) and null presence (2) models, and tests of observed MD model against null climate (1) and null presence (2) models. All four outcomes were considered in evaluating whether there is a significant effect of climate on a species’ distribution

The classic characterisation of SDMs is the use of observed environmental predictor data within a study area to predict observed presence data of a particular species. We use the term ‘observed SDM’ to describe this situation. In order to deal with the aforementioned issues concerning the use of absolute AUC values for SDM evaluation, observed SDMs were assessed against two types of null models, each of which results from the application of an SDM to a combination of observed and null data. This null data takes two forms: (1) null presence data that replicate the spatial pattern in the observed species distribution or (2) null climate data that replicate both the spatial pattern in each observed climate variable and the relationships between each variable. For each species, a null presence model was built by applying an SDM to null presence data and observed climate data, whilst a null climate model was built by applying an SDM to that species' observed distribution and null climate data (Fig. 2). Species presence data were split 1000 times into training and testing points (60:40 %, respectively; see Additional file 2) to enable the generation of AUC values for observed models and null models. In order to assess the applicability of the observed SDM for each species, we compared its AUC value against the AUC values from 99 null models. The climate signal identified by an observed model was considered to be significant if that model’s median AUC score (from 1000 data splits) was significantly higher than those of 95 of the 99 null models, corresponding to a one-tailed significance level of *p* ≤ 0.05 (see Additional file 2). This evaluation was replicated for each species using both null modelling methods and both SDM techniques (Fig. 2), giving an assessment for four models per species: Maxent null presence, Maxent null climate, MD null presence and MD null climate. Full details of the steps undertaken to generate null species distributions and null climates and evaluate observed models against null models can be found in (Additional file 2: Figure S1), which includes an example null species distribution.

For species where a significant association between predictor variables and species presences was found by model evaluation, models were projected under current climate and future climate projections. All presence data for each tick species were used to train the models for projection in current and future climates. This ensured that the maximum amount of information on the current climate niche of the species was available to the model [54, 55]. Climate suitability maps are presented as averages of Maxent and MD outputs. The similarity between observed and future projected climate suitability for these species was quantified using the Schoener’s *D* statistic which represents the degree of overlap between climate suitability maps, ranging from 0 (no overlap) to 1 (complete overlap) [56, 57]. This comparison was undertaken between 40-year averages of observed climate and those in 2050 and 2098 under four RCPs, using the ENMTools software v1.3 [58].

## Results

### Principal component analysis of climate data

The first three PCs explained 60 %, 24 % and 6 % respectively of the total variation in the 20 climate variables subjected to PCA within the western Palearctic. There were two clear groups in the variables’ contributions to PC1; all temperature variables had a positive loading, with the exception of temperature seasonality (negligible effect), and the addition of the seasonality of precipitation and saturation deficit during spring and summer. All 7 remaining precipitation variables had negative loadings. This is likely to reflect the effects of latitude and, to a lesser degree, altitude; regions at high latitude and/or altitude experience relatively lower temperatures and saturation deficit, and large amounts of precipitation with relatively low seasonal variation. In contrast, the seasonality and annual range of temperatures contribute most to PC2, with high positive loadings, whereas all the other variables contribute negatively. This appears to demonstrate the effects of continentality, with coastal areas’ temperature fluctuations strongly moderated by the sea. Substantial negative loadings were winter extremes of temperature and rainfall. Mean temperature of the wettest quarter made the largest negative contribution to PC3, whilst the annual range of temperatures was the highest positive loading. Detailed PCA results are provided in Additional file 3.

### Comparison of SDMs for tick species against null models

Seven out of eight tick species demonstrated significant associations between predictor variables and species presences. For six of these species, median AUC values across 1000 data splits for observed SDMs were significantly higher than at least 95 of the corresponding null models (Table 1). This was consistent across both null modelling methods (null climate and null species distribution) and both SDM techniques (Maxent and MD). The performance of the SDMs of the seventh species, *I. ricinus* (as measured by median AUC), was significantly better than ≥98 models based on null species distributions (Maxent and MD), and 96 of the null climate models (MD). Median AUC of the *I. ricinus* Maxent SDM was greater than all 99 of the corresponding models based on null climate, and significantly higher than 94 of them.

**Table 1:** Performance of observed SDMs based on observed tick distributions and climate compared to null models

Tick species	Maxent null climate	Maxent null species distribution	Mahalanobis distance null climate	Mahalanobis distance null species distribution
*Dermacentor marginatus*	96	98	95	99
*Haemaphysalis punctata*	96	98	98	99
*Haemaphysalis sulcata*	96	96	98	99
*Hyalomma lusitanicum*	88	64	94	72
*Hyalomma marginatum*	95	99	96	98
*Ixodes ricinus*	94	98	96	99
*Rhipicephalus annulatus*	96	95	98	96
*Rhipicephalus bursa*	95	98	95	99

Values indicate the number of null models (*n* = 99) whose median AUC scores were significantly exceeded by those of observed models (1000 random 60:40 training:testing data splits per model). Significant differences identified using *post hoc* multiple comparison tests with Bonferroni corrected critical values from the t distribution. Attainment by an observed model of a median AUC score significantly higher than those of 95 null models was deemed to indicate a significant relationship between the climate predictor variables and the distribution of the tick species. Underlined values indicate that no significant relationship was identified for that species’ observed model(s)

Although median AUC values for *Hy. lusitanicum* SDMs exceeded 0.9, they were not significantly higher than 95 of the null models, irrespective of SDM technique or null modelling method employed. Consequently, it cannot be concluded that there is a significant climate signal on the distribution of the species and therefore no further modelling was undertaken for this species.

### Current climate suitability for tick species

**Fig. 3:**
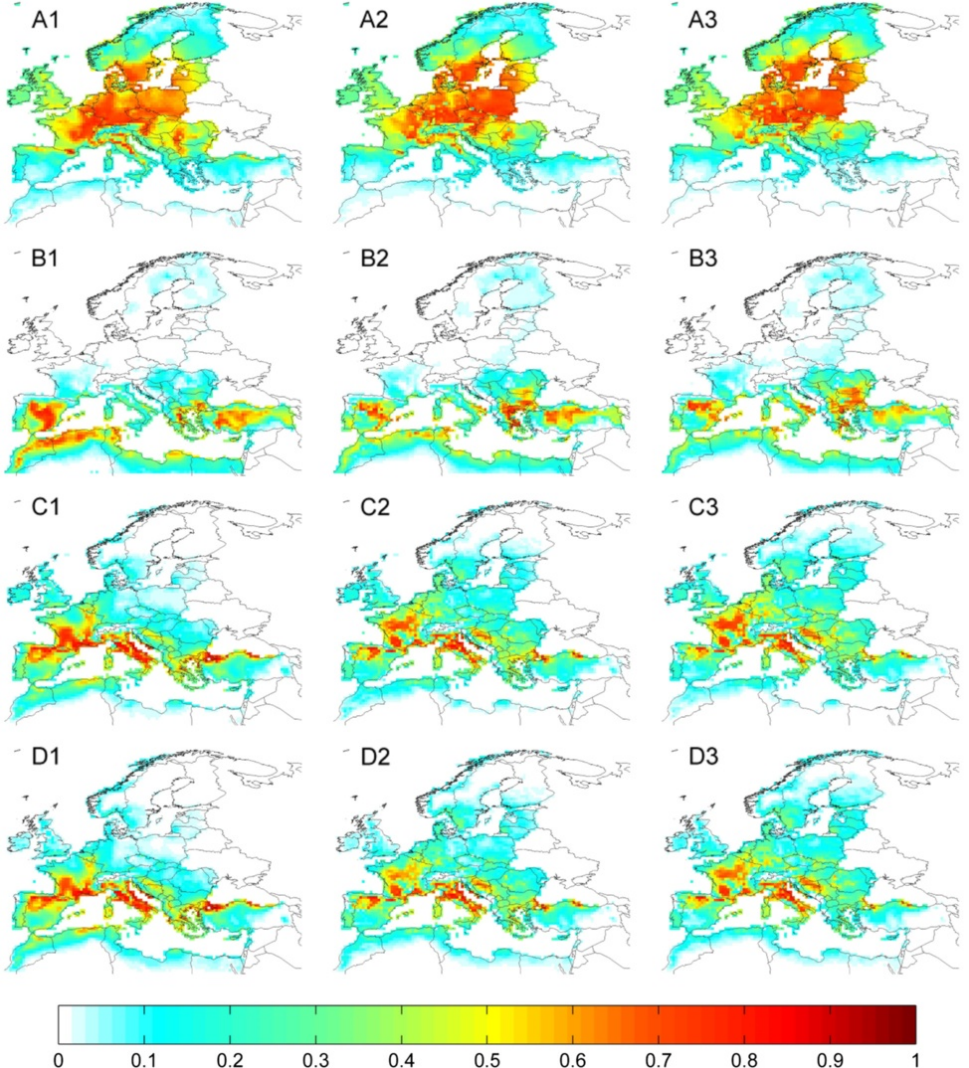
Current and future (RCP 4.5) projected climate suitability for tick species in the western Palearctic. Each row corresponds to a tick species: **a**: *Ixodes ricinus;* **b**: *Rhipicephalus annulatus*; **c**: *Dermacentor marginatus*; **d**: *Haemaphysalis punctata.* Columns correspond to 40-year temporal averages up to and including: 1: 2010; 2: 2050; 3: 2098. Figures in column 1 represent the average suitability derived from Maxent and MD SDMs based on observed climate; columns 2 and 3 contain suitability averaged across Maxent and MD SDMs produced from four GCMs following RCP 4.5. Values range from 0 (unsuitable) to 1 (highly suitable)

**Fig. 4:**
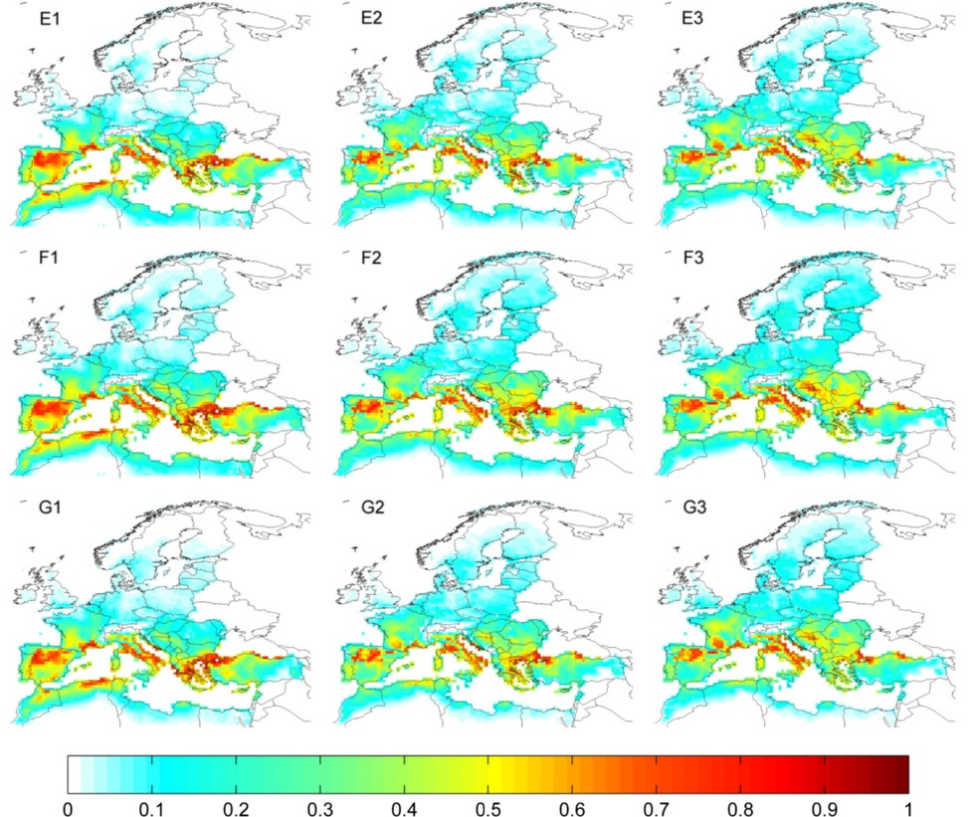
Current and future (RCP 4.5) projected climate suitability for tick species in the western Palearctic. Each row corresponds to a tick species: **e**: *Haemaphysalis sulcata*; **f**: *Hyalomma marginatum*; **g**: *Rhipicephalus bursa.*Columns correspond to 40-year temporal averages up to and including: 1: 2010; 2: 2050; 3: 2098. Figures in column 1 represent the average suitability derived from Maxent and MD SDMs based on observed climate; columns 2 and 3 contain suitability averaged across Maxent and MD SDMs produced from four GCMs following RCP 4.5. Values range from 0 (unsuitable) to 1 (highly suitable)

For species where a significant relationship between their observed distribution and climate was identified (Table 1), suitability maps were produced from the averaged outputs of Maxent and MD models (Figs. 3 and 4, column 1). These indicate that large areas of the western Palearctic are currently climatically suitable for one or more species of tick. Models show that *I. ricinus* is the species with the largest climatically suitable area (Fig. 3.A.1), reflecting its considerable geographic distribution (Fig. 1.F). A large area of high climatic suitability for the species covers much of central Europe (from France eastwards to Poland), with moderate suitability extending into the Baltic states, southern Scandinavia and the British Isles. The southern edge of the suitable area lies across northern Iberia in the west and northern Greece and Turkey in the east, with negligible suitability around the northern Mediterranean coast. The area of climatic suitability for *R. annulatus*, in contrast, occurs in the south of the western Palearctic, with highly suitable areas in northern Africa, Spain and Turkey, but little suitability north of Iberia (Fig. 3.B.1).Suitable areas for the remaining species (*D. marginatus, H. punctata, H. sulcata, Hy. marginatum* and *R. bursa*) occur largely around the Mediterranean, extending into central Europe to varying degrees (Figs. 3.C-D.1 and 4.E-G.1, respectively). Suitable areas for *D. marginatus* and *H. punctata* occur in central France and in the northern Mediterranean, whereas predicted suitable climate for the remaining species is more widespread throughout the Mediterranean region.

### Future trends in climatic suitability for tick species

For all species where a significant climate signal was detected, the general future trend across the four RCPs was for a northward shift in climatically suitable areas. The magnitude of this shift was most pronounced for RCP8.5 climate, and relatively minor for that of RCP2.6. Averaged climate suitability results are presented for RCP4.5 (Figs. 3 and 4, columns 2 & 3). Under this pathway, the central area of highly suitable climate for *I. ricinus* shifts northward slightly by 2050 (Fig. 3.A.2), and more markedly by 2098 (Fig. 3.A.3). Suitability increases considerably in Poland, the Baltic states and southern Finland, whilst remaining high in Germany. Current areas of high suitability in France, the Pyrenees, northern Italy and western Romania are projected to become gradually less suitable by the end of the 21st century. The overall climatically suitable range of *R. annulatus* is not projected to shift considerably under RCP4.5, although there is a tendency within this range for the highest areas of suitability to shift further north by 2098, from northern Africa into northern Spain and out of Turkey into Bulgaria and Romania (Fig. 3.B.2–3). Highly suitable areas for *D. marginatus* (Fig. 3.C.2–3) and *H. punctata* (Fig. 3.D.2-3) shift northward within Italy and from northern Iberia into central France, with some reduction of suitable areas along the lowland southern Black Sea coast and a moderate increase in Croatia. The remaining species (*H. sulcata, Hy. marginatum* and *R. bursa*) present similar tendencies (Fig. 4.E-G.2–33, respectively); a reduction of observed suitability in north Africa and southern Iberia, and modest incremental northward increases in suitability across France and the Balkans. Amongst these species, suitability for *H. sulcata* increases the most.

**Fig. 5:**
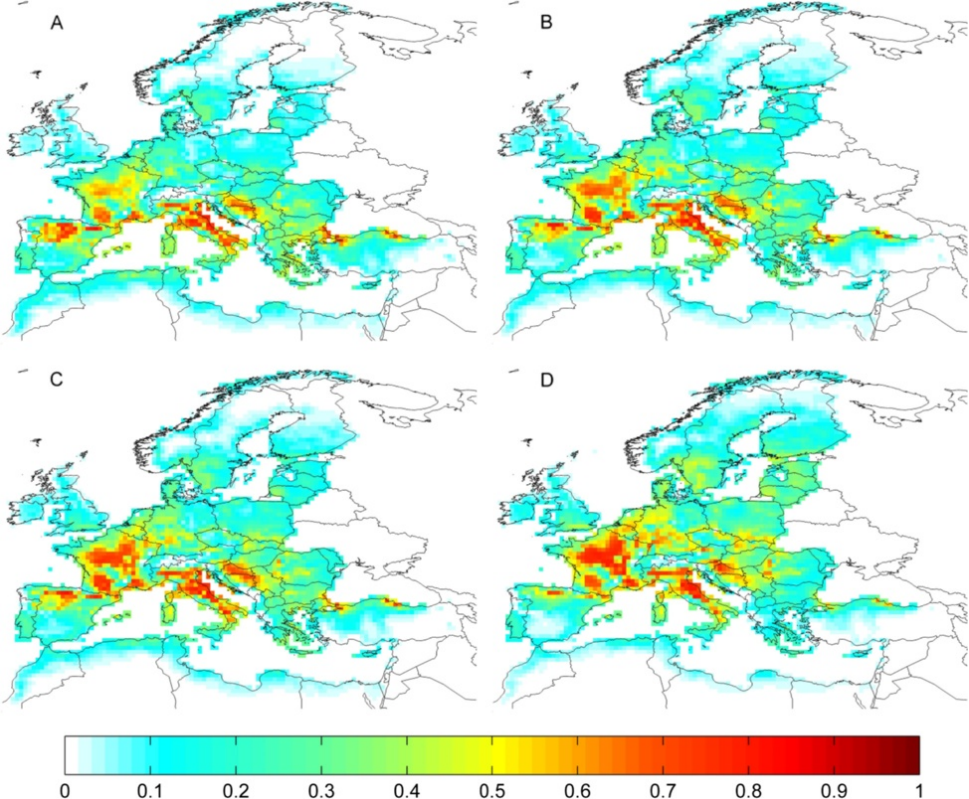
Future projected climate suitability for *Haemaphysalis punctata* in the western Palearctic under four RCPs. Average suitability from Maxent and MD SDMs using climate data averaged over 40 years up to and including 2098, as simulated by 4 GCMs. **a**: RCP 2.6; **b**: RCP 4.5; **c**: RCP 6.0; **d**: RCP 8.5. Values range from 0 (unsuitable) to 1 (highly suitable)

A pattern illustrative of the effects of different RCP climates on suitability is presented in Fig. 5 for *H. punctata*, with the degree of change in suitability increasing commensurate with the degree of radiative forcing projected. Models under observed climate predicted high suitability in northern Iberia, southern France, Italy and the southern Black Sea coast (Fig. 3.D.1) for this species; by the end of the 21st century, even under the most conservative RCP 2.6, suitability is projected to increase slightly in Croatia and central France, whilst there is a modest reduction in suitability in southern areas (Fig. 5.A). There is a greater increase in suitability in central France under RCP 4.5 (Fig. 5.B), and diminished suitability along the southern coast of the Mediterranean. Under RCP 6.0 climate, large areas of central France are projected to be even more highly suitable, in addition to northern Italy (Fig. 5.C), whilst RCP 8.5 climate renders suitability in these areas and Croatia even higher, moderate suitability across the Benelux countries and western Germany, and low suitability extends even to southern Scandinavia. This pathway of continued climate change without stabilization is projected to result in considerable reduction of suitability for *H. punctata* along southern Mediterranean and Black Sea coasts (Fig. 5.D). (Additional file 4: Table S2) illustrates the decreasing similarity between observed and future climate suitabilities with increasingly extreme RCP climate, as Schoener’s *D* statistic of similarity [56, 57] is considerably reduced by 2098 for higher RCPs. Projected climate suitability for the other species under RCPs 2.6, 6.0 and 8.5 can be found in Additional file 4: Figures S3-S8.

### Inter-SDM comparison

**Fig. 6:**
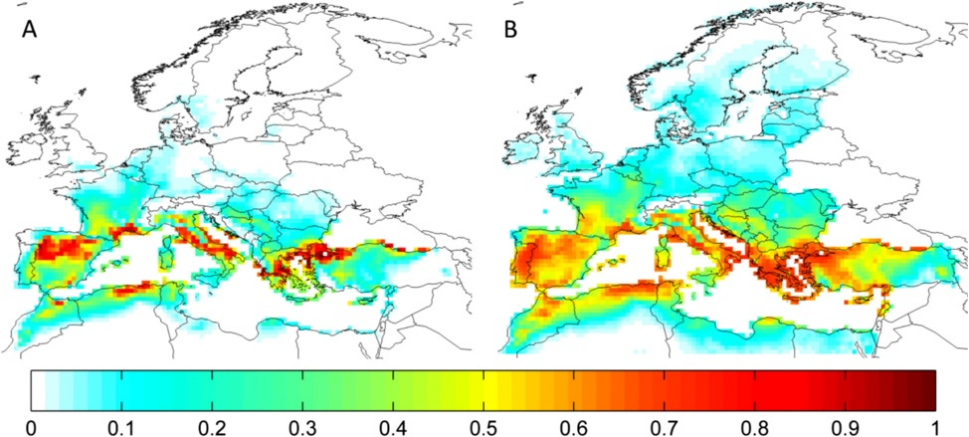
Comparison of Mahalanobis Distance (**a**) and Maxent (**b**) species distribution predictions for *Rhipicephalus bursa* with observed climate. Models used climate data averaged over 40 years up to and including 2010. Values range from 0 (unsuitable) to 1 (highly suitable)

There was considerable agreement between Maxent and MD SDMs on central areas of high suitability for each species. The models consistently concurred on these core areas across both observed and projected climates. In general, however, the MD method predicted a more restricted geographic range of climatically suitable areas, with relatively steeper gradients from high to low suitability than those evident in Maxent predictions (Fig. 6). Consequently, some observed species presences occur in areas assessed by the MD model as presenting very low climatic suitability.

## Discussion

This study applies, for the first time in our knowledge, null model methodologies to test the strength of the association between climate and disease vector distributions. Previous comparisons of observed SDMs against null models indicate that spatial patterns in observed data may inflate SDM performance metrics, inferring stronger associations in observed models than may actually be present [13, 14]. In order to robustly assess the strength of climate signals in tick distributions, we therefore tested observed Maxent and MD SDMs against null models. These used null data retaining a measure of the spatial pattern evident in observed data, either as null climate predictors of observed species distributions or as null species distributions predicted by observed climate variables. Consequently, any deterministic relationships between observed predictor and occurrence data were broken in the null models. In the simplest terms, an observed model was required to demonstrate significantly higher predictive performance than corresponding null models for us to accept that a climate signal was present in a tick species’ distribution. A significant climate signal was identified in SDMs of seven out of eight tick species across the western Palearctic after robust challenge by null models, highlighting the applicability of these models for identifying currently suitable climate – and consequently for projecting suitability under future climate change.

*Hy. lusitanicum* was the only modelled species that did not reveal a climatic signal in its distribution that was significantly stronger than those produced from null species distributions or climates. Clearly, climate is one amongst a plethora of factors which influence the distribution of tick species [2], and although it is frequently held to be the primary influence on distribution [55, 59], this is not always the case; for example, host and habitat distribution play an important role (see [2] and [19] for comprehensive reviews of tick ecology). Our climate-driven SDMs did not take host or habitat distribution into account, owing to the lack of detailed information on current and especially future distributions. Some authors have attributed the restricted distribution of *Hy. lusitanicum* to its strict biotic niche [22], as larvae and nymphs have been reported to specifically parasitise the European rabbit *Oryctolagus cuniculus* in its burrow, and the tick species is reportedly not present where the host is absent [60]. However, immature stages have also been recorded on other hosts, with the most likely being various small mammals, not solely *O. cuniculus* [61]. The species distribution in the dataset used in this study [33] corresponds well with that described by Apanaskevich *et al.* (2008) from historic literature and recent samples [61], whilst acknowledging that the species is relatively poorly studied. Even though the observed SDMs for *Hy. lusitanicum* failed to statistically outperform the null models, they returned very high AUC values (medians range between 0.9073 and 0.9091) which would indicate excellent predictive performance were the assessment of model success made solely on absolute AUC values [5]. However, it can be seen from Fig. 1.D that the observed distribution for this tick species is amongst the most geographically restricted of all those in this study, and such restricted species distributions frequently result in inflated AUC values [8, 11]. Comparison of observed SDMs against null models failed to show a significant climate signal in this species’ distribution, despite the high AUC scores obtained by observed models; this suggests that factors other than climate are the main driver on the distribution of this species.

SDMs aim to characterise the multi-dimensional ecological space of a species, and there are several important considerations when employing these modelling techniques. Firstly, species presence data are assumed to be representative of a species’ actual distribution and the complete range of ecological conditions within which a species is found [62]. There are numerous reasons why this may not be the case. Data are frequently gathered from surveys which often focus on a particular geographic area or species and are not systematic [33]; this introduces bias and results in potentially incomplete datasets. For example, there is an acknowledged lack of records of *D. reticulatus* in parts of its known range in the dataset used in this study [33], and it was therefore excluded from modelling. Misidentification of specimens may also contribute to unreliable data, particularly given the difficulties of morphological identification and debated taxonomy of some ticks [63]; this is a particular problem for the *Hyalomma* genus [64]. Whilst we cannot be certain that these issues do not affect the dataset used in this study, it has been compiled from a broad range of sources, over a long temporal period and we have excluded species with recognised data inadequacies. Secondly, it should be recognised that whilst climate-based SDMs attempt to characterise a species’ fundamental niche, observed species presence data represent the species’ realised niche (see [62]), as non-climatic influences such as habitat and host distributions and historical dispersal patterns may have excluded them from climatically-suitable areas. Where these influences exclude the species from a portion of its observed fundamental niche, this may render projected climate suitability a conservative approximation of suitable climatic conditions. In addition, all climatically suitable areas identified by SDMs may not be inhabited by ticks, as their distribution within these areas may be moderated by the aforementioned non-climatic factors. Thirdly, there is an inherent assumption in species distribution modelling that individuals of a species react homogeneously to the predictor variables which drive the model [55]. However, sub-populations of a species frequently exhibit adaptation to local conditions, violating this assumption of stationarity [65]; this is particularly likely for widespread species (see [10]). There is evidence for *I. ricinus* of distinct ecological preferences amongst sub-populations (clades) [66], genetic divergence [67] and phenotypic differences [68]. Such local adaptation is likely to reduce the accuracy of SDMs [10]; whilst it would be preferable to model each clade separately, using biologically relevant data partitions (*e.g.* [69]), these clades are not static [66], and detailed knowledge of their future distributions is lacking. We have therefore modelled the whole western Palearctic population of *I. ricinus* as a single entity. Nevertheless, three out of four of our assessments presented here have identified a significant effect of climate on the distribution of *I. ricinus*, as observed models performed significantly better than null models. The remaining observed model obtained a higher AUC than 94 of the 99 Maxent null climate models, thereby only marginally failing to reach significance.

Uncertainty arises in species distribution modelling from the choice of SDM, GCM and climate change scenario [49]. In order to minimise the effects of this, we have presented suitability averaged across Maxent and MD SDMs, as model choice has been shown to be the largest source of uncertainty [49]. Maxent modelling has been found to predict a larger extent of suitable area, with a more gradual gradient from high to low suitability, than the relatively more restricted spatial predictions of MD modelling [70]. Our results corroborate this finding; Fig. 6 illustrates the difference in the predictions of suitability for *R. bursa.* Nonetheless, the two SDMs broadly concurred on core areas of high suitability, with disparity mainly confined to the margins where the species may be at the periphery of their ecological space. GCMs account for an increasing proportion of variability in SDM predictions with increasing distance in the future [49], and are also known to contain biases [46]; this uncertainty has been reduced by generating future projections which have been averaged across the 4 GCMs used in this study, and these are presented separately for each RCP (Fig. 5; see also in Additional file 4: Figures S3-8).

Apparent shifts in tick distributions have been documented in recent decades from multiple sources, and there has evidently been a spread of some species to both higher latitudes and altitudes; for example *I. ricinus* in Sweden [71] and the Czech Republic [72], respectively. Climatic change has been strongly implicated in these distributional changes. The western Palearctic region is projected to experience increased future temperatures, particularly in southern summers and northern winters, in addition to an increase in precipitation in the north and a reduction in the south [73] with concomitant opposite trends in saturation deficit. These changes are likely to reduce the limitations on tick distributions imposed by low temperatures at high altitude and latitude, whilst increasing restrictions on southern margins where increased saturation deficit during the spring and summer months will further restrict species’ ranges [20, 22]. This has been projected to result in a northward shift of climatically suitable conditions for ticks by previous SDMs [22, 23] as well as process-based population models of historic and potential future shifts [74, 75].

Climatically suitable areas for species currently exhibiting Mediterranean distributions are generally projected by this study to shift northwards under future climate change (Figs. 3 and 4), with *D. marginatus* and *H. punctata* experiencing the greatest shift (Additional file 4: Table S2), particularly within France and the Balkans (Fig. 3.C-D.1-3). These regions were also projected by Estrada-Peña and Venzal’s model [22] to become more suitable for the former species under drier and hotter climate. *R. annulatus*, conversely, has the most constrained range of suitable climate of all the species modelled; suitable areas are projected to shift only moderately northwards whilst current high climate suitability in northern Africa and southern Spain will be considerably reduced in the future. Previous modelling [22] forecasted minimal effects of changes in monthly rainfall on *R. annulatus*, but species records from Turkey and Spain were largely absent from that study and projections diverge markedly in these regions. Future climate suitability for *R. bursa* is projected to follow a similar trend to *R. annulatus*, with a northward shift (as projected by [22]) and southern decline reflecting the hygrophilic nature of the species, which may already be declining in parts of northern Africa [76]. A literature search revealed no modelling of the distribution of *H. punctata* or *H. sulcata* in relation to climate. Our projection of a future northward shift in suitable climate for *Hy. marginatum* corresponds well with projections from a correlational SDM [22] in addition to a population model [74] for this species. As the most widespread species in the western Palearctic, *I. ricinus* has been subject to considerable modelling effort. Predictions of current climate suitability (Fig. 3.A.1) incorporate much of the known distribution of *I. ricinus* (Fig. 1.F), and overlap substantially with areas of high performance rates predicted by a process-driven model constructed using temperature, saturation deficit and daylight [28]. Our current predictions are further corroborated by predicted suitability from a Maxent model based on remotely sensed variables [26], which additionally predicted increased climate suitability in southern Scandinavia, the Balkans and eastern Europe in the early 21st century. This supports our projections of increased future suitability in these regions (Fig. 3.A.2-3), whilst further indication of northward range expansion is provided by projections for Scandinavia based on vegetation phenology [77].

The results of this study have been shown to be commensurate with many previous publications which projected northward shifts in climate suitable for tick species. However, this is the first time that a null modelling approach, using latest AR5 climate projections, has been employed consistently to eight tick species simultaneously. This affords considerable confidence that our findings result from climate drivers of distribution and are not simply an artefact of autocorrelation in the spatial datasets used. Consequently, this supersedes the most prominent previous multi-species tick modelling analysis [22] which was undertaken before such robust assessments of SDM performance against null models were developed [13, 14], and did not make use of internationally-recognised climate scenarios. Many authors have developed bespoke SDMs for individual tick species, but comparison of projections under climate change from individual models is challenging, due to the numerous sources of variation in the projections, whereas our application of two SDM approaches to eight species permits a greater comparability of the overall trends in future climatic suitability.

## Conclusion

A changing climate during the 21st century is likely to pose numerous significant risks and challenges to society. One of these is a projected change in the climate suitable for parasites and pathogens. This paper highlights a future geographic shift in the climate that is currently suitable for several disease-carrying tick species. This conclusion was reached after using some of the most demanding performance-measuring methods available for use with SDMs and constitutes a much more robust statistical evaluation than is the norm in the field. The consistent application of this methodology to a high quality tick presence dataset offers a valuable insight into the potential for future changes in the climatically suitable areas for these tick species.

## Additional files

Additional file 1:
**Maxent model fitting and selection**. Procedure followed to generate Maxent models and assess their fit; characteristics of selected models. (PDF 321 kb) Additional file 2:
**Supplementary methods for null data generation and evaluation of observed models against null models.** Detailed description of the methods used to generate null data which retained spatial patterns evident in observed data, followed by information on null model construction and testing of observed models against null models. Includes Figure S1: Example of null species distribution data generated for *Haemaphysalis punctata*. A: observed species distribution; B: example null species distribution; C: semivariogram showing the relationship between semivariance and distance for observed species data (blue line) and 99 null species distributions (grey lines). (PDF 249 kb) Additional file 3:
**PCA results. Table S1: Loadings of Principal Component Analysis of western Palearctic climate.** 20 climate variables averaged over 40 years (1971–2010); data obtained from Climate Research Unit time series (version 3.22). Figure S2: Mapped Principal Component (PC) scores in western Palearctic. Results from PCA of 40-year averages (1971–2010) of 20 observed climate variables. A: PC1; B: PC2; C: PC3. (PDF 248 kb) Additional file 4:
**Projected current and future climate suitability under RCP 2.6, 6.0 & 8.5. Figures S3-S8 & Table S2. Figure S3.** Current and future (RCP 2.6) projected climate suitability for tick species in the western Palearctic. Each row corresponds to a tick species: A: *Ixodes ricinus;* B: *Rhipicephalus annulatus*; C: *Dermacentor marginatus*; D: *Haemaphysalis punctata.* Columns correspond to 40-year temporal averages up to and including: 1: 2010; 2: 2050; 3: 2098. Figures in column 1 represent the average suitability derived from Maxent and MD SDMs based on observed climate; columns 2 and 3 contain suitability averaged across Maxent and MD SDMs produced from four GCMs following RCP 2.6. Values range from 0 (unsuitable) to 1 (highly suitable). Figure S4. Current and future (RCP 2.6) projected climate suitability for tick species in the western Palearctic. Each row corresponds to a tick species: E: *Haemaphysalis sulcata*; F: *Hyalomma marginatum*; G: *Rhipicephalus bursa.* Columns correspond to 40-year temporal averages up to and including: 1: 2010; 2: 2050; 3: 2098. Figures in column 1 represent the average suitability derived from Maxent and MD SDMs based on observed climate; columns 2 and 3 contain suitability averaged across Maxent and MD SDMs produced from four GCMs following RCP 2.6. Values range from 0 (unsuitable) to 1 (highly suitable). Figure S5. Current and future (RCP 6.0) projected climate suitability for tick species in the western Palearctic. Each row corresponds to a tick species: A: *Ixodes ricinus;* B: *Rhipicephalus annulatus*; C: *Dermacentor marginatus*; D: *Haemaphysalis punctata*. Columns correspond to 40-year temporal averages up to and including: 1: 2010; 2: 2050; 3: 2098. Figures in column 1 represent the average suitability derived from Maxent and MD SDMs based on observed climate; columns 2 and 3 contain suitability averaged across Maxent and MD SDMs produced from four GCMs following RCP 6.0. Values range from 0 (unsuitable) to 1 (highly suitable). Figure S6. Current and future (RCP 6.0) projected climate suitability for tick species in the western Palearctic. Each row corresponds to a tick species: E: *Haemaphysalis sulcata*; F: *Hyalomma marginatum*; G: *Rhipicephalus bursa.* Columns correspond to 40-year temporal averages up to and including: 1: 2010; 2: 2050; 3: 2098. Figures in column 1 represent the average suitability derived from Maxent and MD SDMs based on observed climate; columns 2 and 3 contain suitability averaged across Maxent and MD SDMs produced from four GCMs following RCP 6.0. Values range from 0 (unsuitable) to 1 (highly suitable). Figure S7. Current and future (RCP 8.5) projected climate suitability for tick species in the western Palearctic. Each row corresponds to a tick species: A: *Ixodes ricinus;* B: *Rhipicephalus annulatus*; C: *Dermacentor marginatus*; D: *Haemaphysalis punctata.* Columns correspond to 40-year temporal averages up to and including: 1: 2010; 2: 2050; 3: 2098. Figures in column 1 represent the average suitability derived from Maxent and MD SDMs based on observed climate; columns 2 and 3 contain suitability averaged across Maxent and MD SDMs produced from four GCMs following RCP 8.5. Values range from 0 (unsuitable) to 1 (highly suitable). Figure S8. Current and future (RCP 8.5) projected climate suitability for tick species in the western Palearctic. Each row corresponds to a tick species: E: *Haemaphysalis sulcata*; F: *Hyalomma marginatum*; G: *Rhipicephalus bursa.* Columns correspond to 40-year temporal averages up to and including: 1: 2010; 2: 2050; 3: 2098. Figures in column 1 represent the average suitability derived from Maxent and MD SDMs based on observed climate; columns 2 and 3 contain suitability averaged across Maxent and MD SDMs produced from four GCMs following RCP 8.5. Values range from 0 (unsuitable) to 1 (highly suitable). Table S2: Similarity between current and future projected climate suitability for seven tick species. Schoener's *D* statistic represents the degree of overlap between climate suitability maps, ranging from 0 (no overlap) to 1 (complete overlap). Smallest values therefore indicate least overlap and so the greatest change between current and future projections of climate suitability. Average climate suitability produced by Maxent and MD SDMs for the 40-year period up to and including 2010 has been compared with climate suitability averaged across both SDMs over future 40-year periods up to and including 2050 and 2098 under all four RCP climates. This analysis was undertaken using ENMTools software v1.3. (PDF 1968 kb)

## Abbreviations

AR5: Fifth Annual Report of the Intergovernmental Panel for Climate Change
AUC: Area under the curve
GCM: General circulation model
MD: Mahalanobis distance
PCA: Principal component analysis
PC: Principal component
RCP: Representative concentration pathway
SDM: Species distribution model

## References

[1] Jongejan F, Uilenberg G (2004). The global importance of ticks. Parasitology.

[2] Estrada-Peña A, de la Fuente J (2014). The ecology of ticks and epidemiology of tick-borne viral diseases. Antiviral Res..

[3] Elith J, Leathwick JR (2009). Species distribution models: ecological explanation and prediction across space and time. Annu Rev Ecol Evol S..

[4] Fielding AH, Bell JF (1997). A review of methods for the assessment of prediction errors in conservation presence/absence models. Environ Conserv.

[5] Araujo MB, Pearson RG, Thuiller W, Erhard M (2005). Validation of species-climate impact models under climate change. Global Change Biol..

[6] Legendre P, Legendre L (1998). Numerical Ecology. Developments in environmental modelling.

[7] Segurado P, Araujo MB, Kunin WE (2006). Consequences of spatial autocorrelation for niche-based models. J Appl Ecol..

[8] Lobo JM, Jimenez-Valverde A, Real R (2008). AUC: a misleading measure of the performance of predictive distribution models. Global Ecol Biogeogr.

[9] Elith J, Graham CH, Anderson RP, Dudik M, Ferrier S, Guisan A (2006). Novel methods improve prediction of species' distributions from occurrence data. Ecography.

[10] Hernandez PA, Graham CH, Master LL, Albert DL (2006). The effect of sample size and species characteristics on performance of different species distribution modeling methods. Ecography.

[11] Jimenez-Valverde A, Lobo JM, Hortal J (2008). Not as good as they seem: the importance of concepts in species distribution modelling. Divers Distrib.

[12] Veloz SD (2009). Spatially autocorrelated sampling falsely inflates measures of accuracy for presence-only niche models. J Biogeogr.

[13] Beale CM, Lennon JJ, Gimona A (2008). Opening the climate envelope reveals no macroscale associations with climate in European birds. P Natl Acad Sci USA.

[14] Chapman DS (2010). Weak climatic associations among British plant distributions. Global Ecol Biogeogr.

[15] Lauzeral C, Grenouillet G, Brosse S (2012). Dealing with noisy absences to optimize species distribution models: An iterative ensemble modelling approach. PLoS One.

[16] Algar AC, Mahler DL, Glor RE, Losos JB (2013). Niche incumbency, dispersal limitation and climate shape geographical distributions in a species-rich island adaptive radiation. Global Ecol Biogeogr.

[17] Austin M (2007). Species distribution models and ecological theory: A critical assessment and some possible new approaches. Ecol Model.

[18] Ostfeld RS, Brunner JL. Climate change and *Ixodes* tick-borne diseases of humans. Philos Trans R Soc Lond, Ser B: Biol Sci. 2015; 370(1665). doi:10.1098/rstb.2014.0051. 10.1098/rstb.2014.0051 PMC4342967 25688022

[19] Pfäffle M, Littwin N, Muders SV, Petney TN (2013). The ecology of tick-borne diseases. Int J Parasit.

[20] Gray JS, Dautel H, Estrada-Peña A, Kahl O, Lindgren E (2009). Effects of climate change on ticks and tick-borne diseases in Europe. Interdiscip Perspect Infect Dis..

[21] Medlock JM, Hansford KM, Bormane A, Derdakova M, Estrada-Peña A, George J-C (2013). Driving forces for changes in geographical distribution of *Ixodes ricinus* ticks in Europe. Parasites Vectors..

[22] Estrada-Peña A, Venzal JM (2007). Climate niches of tick species in the Mediterranean region: modeling of occurrence data, distributional constraints, and impact of climate change. J Med Entomol.

[23] Porretta D, Mastrantonio V, Amendolia S, Gaiarsa S, Epis S, Genchi C (2013). Effects of global changes on the climatic niche of the tick *Ixodes ricinus* inferred by species distribution modelling. Parasites Vectors..

[24] Alonso-Carné J, Garcia-Martín A, Estrada-Peña A (2015). Assessing the statistical relationships among water-derived climate variables, rainfall, and remotely sensed features of vegetation: implications for evaluating the habitat of ticks. Exp Appl Acarol.

[25] Perret JL, Guerin PM, Diehl PA, Vlimant M, Gern L (2003). Darkness induces mobility, and saturation deficit limits questing duration, in the tick *Ixodes ricinus*. J Exp Biol.

[26] Estrada-Peña A, Ayllón N, de la Fuente J (2012). Impact of climate trends on tick-borne pathogen transmission. Front Physiol..

[27] Estrada-Peña A, De la Fuente J, Latapia T, Ortega C (2015). The impact of climate trends on a tick affecting public health: a retrospective modeling approach for *Hyalomma marginatum* (Ixodidae). PLoS One.

[28] Estrada-Peña A, Estrada-Sanchez D (2014). Deconstructing *Ixodes ricinus*: a partial matrix model allowing mapping of tick development, mortality and activity rates. Med Vet Entomol.

[29] Perret JL, Guigoz E, Rais O, Gern L (2000). Influence of saturation deficit and temperature on *Ixodes ricinus* tick questing activity in a Lyme borreliosis-endemic area (Switzerland). Parasitol Res.

[30] Milne A (1950). The ecology of the sheep tick, Ixodes-Ricinus L - microhabitat economy of the adult tick. Parasitol.

[31] Gray JS (1982). The development and questing activity of the tick *Ixodes ricinus* under field conditions in Ireland. Bull Entomol Res..

[32] Randolph SE (2002). Predicting the risk of tick-borne diseases. Int J Med Microbiol..

[33] Estrada-Peña A, Farkas R, Jaenson TG, Koenen F, Madder M, Pascucci I (2013). Association of environmental traits with the geographic ranges of ticks (Acari: Ixodidae) of medical and veterinary importance in the western Palearctic. A digital data set. Exp Appl Acarol.

[34] Gray J, Dantas-Torres F, Estrada-Peña A, Levin M (2013). Systematics and ecology of the brown dog tick, *Rhipicephalus sanguineus*. Ticks Tick-Borne Dis..

[35] IPCC (2014). Climate Change 2014: Synthesis Report. Contribution of Working Groups I, II and III to the Fifth Assessment Report of the Intergovernmental Panel on Climate Change Geneva.

[36] Hijmans RJ, Cameron SE, Parra JL, Jones PG, Jarvis A (2005). Very high resolution interpolated climate surfaces for global land areas. Int J Climatol.

[37] Busby JR (1991). BIOCLIM—a bioclimatic analysis and prediction tool. Plant Prot Q..

[38] Mitchell TD, Carter TR, Jones PD, Hulme M, New M (2004). A comprehensive set of high-resolution grids of monthly climate for Europe and the globe: the observed record (1901–2000) and 16 scenarios (2001–2100).

[39] New M, Hulme M, Jones P (2000). Representing Twentieth century space-time climate variability. Part II: Development of 1901–96 monthly grids of terrestrial surface climate. J Clim.

[40] Allen RG, Pereira LS, Raes D, Smith M (1998). Crop evapotranspiration - Guidelines for computing crop water requirements - FAO Irrigation and drainage paper 56. FAO Irrigation and Drainage Papers.

[41] Estrada-Peña A, Martinez JM, Acedo CS, Quilez J, del Cacho D (2004). Phenology of the tick, *Ixodes ricinus*, in its southern distribution range (central Spain). Med Vet Entomol..

[42] Milne A (1945). The ecology of the sheep tick, *Ixodes ricinus*. The seasonal activity in Britain with particular reference to northern England. Parasitol.

[43] Estrada-Pena A, Martinez Aviles M, Munoz Reoyo MJ (2011). A population model to describe the distribution and seasonal dynamics of the tick *Hyalomma marginatum* in the Mediterranean Basin. Transbound Emerg Dis.

[44] Hempel S, Frieler K, Warszawski L, Schewe J, Piontek F (2013). A trend-preserving bias correction: the ISI-MIP approach. Earth Syst Dynam.

[45] Moss RH, Edmonds JA, Hibbard KA, Manning MR, Rose SK, van Vuuren DP (2010). The next generation of scenarios for climate change research and assessment. Nature.

[46] Masaki Y, Hanasaki N, Takahashi K, Hijioka Y (2015). Propagation of biases in humidity in the estimation of global irrigational water. Earth Syst Dynam Discuss.

[47] Jolliffe IT (2002). Principal Component Analysis. 2 ed. Springer Series in Statistics.

[48] Segurado P, Araujo MB (2004). An evaluation of methods for modelling species distributions. J Biogeogr.

[49] Buisson L, Thuiller W, Casajus N, Lek S, Grenouillet G (2010). Uncertainty in ensemble forecasting of species distribution. Global Change Biol.

[50] Elith J, Kearney M, Phillips S (2010). The art of modelling range-shifting species. Methods Ecol Evol.

[51] Phillips SJ, Dudik M (2008). Modeling of species distributions with Maxent: new extensions and a comprehensive evaluation. Ecography.

[52] Farber O, Kadmon R (2003). Assessment of alternative approaches for bioclimatic modeling with special emphasis on the Mahalanobis distance. Ecol Model.

[53] Clark JD, Dunn JE, Smith KG (1993). A multivariate model of female black bear habitat use for a geographic information system. J Wildlife Manage.

[54] Thuiller W, Brotons L, Araujo MB, Lavorel S (2004). Effects of restricting environmental range of data to project current and future species distributions. Ecography.

[55] Estrada-Peña A, Estrada-Sanchez A, Estrada-Sanchez D (2015). Methodological caveats in the environmental modelling and projections of climate niche for ticks, with examples for *Ixodes ricinus* (Ixodidae). Vet Parasitol.

[56] Schoener TW (1968). The Anolis lizards of Bimini: resource partitioning in a complex fauna. Ecology.

[57] Warren DL, Glor RE, Turelli M (2008). Environmental niche equivalency versus conservatism: quantitative approaches to niche evolution. Evolution.

[58] Warren DL, Glor RE, Turelli M (2010). ENMTools: a toolbox for comparative studies of environmental niche models. Ecography.

[59] Cumming GS (2002). Comparing climate and vegetation as limiting factors for species ranges of African ticks. Ecology.

[60] Pérez-Eid C, Cabrita J (2003). La larve et la nymphe de *Hyalomma* (*Hyalomma*) *lusitanicum* Koch, 1844 (Acari: Ixodida): Description morphologique, habitats, hotes. Acarologia.

[61] Apanaskevich DA, Santos-Silva MM, Horak IG (2008). The genus *Hyalomma* Koch, 1844. IV. Redescription of all parasitic stages of *H.* (*Euhyalomma*) *lusitanicum* Koch, 1844 and the adults of *H.* (*E.*) *franchinii* Tonelli Rondelli, 1932 (Acari: Ixodidae) with a first description of its immature stages. Folia Parasitol.

[62] Araujo MB, Guisan A (2006). Five (or so) challenges for species distribution modelling. J Biogeogr.

[63] Estrada-Pena A, Gray JS, Kahl O, Lane RS, Nijhof AM (2013). Research on the ecology of ticks and tick-borne pathogens - methodological principles and caveats. Front Cell Infect Microbiol..

[64] Apanaskevich DA, Horak IG (2008). The genus Hyalomma Koch, 1844: V. Re-evaluation of the taxonomic rank of taxa comprising the *H.* (*Euhyalomma*) *marginatum* Koch complex of species (*Acari* : *Ixodidae*) with redescription of all parasitic stages and notes on biology. Int J Acarol.

[65] Unwin A, Unwin D (1998). Exploratory spatial data analysis with local statistics. J Roy Stat Soc D-Sta..

[66] Estrada-Peña A, Venzal JM, Acedo CS (2006). The tick *Ixodes ricinus*: distribution and climate preferences in the western Palaearctic. Med Vet Entomol..

[67] Noureddine R, Chauvin A, Plantard O (2011). Lack of genetic structure among Eurasian populations of the tick *Ixodes ricinus* contrasts with marked divergence from north African populations. Int J Parasitol.

[68] Estrada-Peña A, Gray JS, Kahl O (1996). Variability in cuticular hydrocarbons and phenotypic discrimination of *Ixodes ricinus* populations (Acarina: Ixodidae) from Europe. Exp Appl Acarol..

[69] Gonzalez SC, Soto-Centeno JA, Reed DL. Population distribution models: species distributions are better modeled using biologically relevant data partitions. BMC Ecol. 2011; 11(20). doi:10.1186/1472-6785-11-20. 10.1186/1472-6785-11-20 PMC3184255 21929792

[70] Hernandez PA, Franke I, Herzog SK, Pacheco V, Paniagua L, Quintana HL (2008). Predicting species distributions in poorly-studied landscapes. Biodivers Conserv.

[71] Jaenson TGT, Jaenson DGE, Eisen L, Petersson E, Lindgren E (2012). Changes in the geographical distribution and abundance of the tick *Ixodes ricinus* during the past 30 years in Sweden. Parasites Vectors..

[72] Danielova V, Rudenko N, Daniel M, Holubova J, Materna J, Golovchenko M (2006). Extension of *Ixodes ricinus* ticks and agents of tick-borne diseases to mountain areas in the Czech Republic. Int J Med Microbiol..

[73] Kovats RS, Valentini R, Bouwer LM EG, Jacob D, Martin E, et al. Europe. In: Barros VR, Field CB, Dokken DJ, Mastrandrea MD, Mach KJ, Bilir TE, et al., editors. Climate Change 2014: Impacts, Adaptation, and Vulnerability. Part B: Regional Aspects. Contribution of Working Group II to the Fifth Assessment Report of the Intergovernmental Panel on Climate Change. Cambridge, United Kingdom and New York, USA: IPCC; 2014.

[74] Estrada-Peña A, Sanchez N, Estrada-Sanchez A (2012). An assessment of the distribution and spread of the tick *Hyalomma marginatum* in the western Palearctic under different climate scenarios. Vector-Borne Zoonotic Dis.

[75] Beugnet F, Kolasinski M, Michelangeli PA, Vienne J, Loukos H (2011). Mathematical modelling of the impact of climatic conditions in France on *Rhipicephalus sanguineus* tick activity and density since 1960. Geospat Health.

[76] Darghouth MA, Bouattour A (2008). Ticks and tick-borne diseases of livestock in North Africa, present state and potential changes in the context of global warming. Livestock and Global Climate Change.

[77] Jaenson TG, Lindgren E (2011). The range of *Ixodes ricinus* and the risk of contracting Lyme borreliosis will increase northwards when the vegetation period becomes longer. Ticks Tick Borne Dis.

