# Supplementary material for: Climate suitability for European ticks: assessing species distribution models against null models and projection under AR5 climate
Source: Parasit Vectors. 2015 Aug 28;8:440. doi: 10.1186/s13071-015-1046-4 (PMC4551698; doi:10.1186/s13071-015-1046-4)
Supplement: Additional file 1: — Maxent model fitting and selection. Procedure followed to generate Maxent models and assess their fit; characteristics of selected models. (PDF 321 kb) [file 13071_2015_1046_MOESM1_ESM.pdf]

Additional file 1: Maxent model fitting and selection

Procedure followed to generate Maxent models and assess their fit; characteristics of selected models.

Supplementary Methods: Maxent model fitting

Maxent does not fit species distribution points directly to the environmental predictor data it is given. Instead, it derives an expanded set of transformed internal features from the environmental predictor data [1]. These features can be linear, quadratic, product, threshold or hinge in form and are designed to capture the different responses of species to their environment [2]. The Maxent software decides which features are available to the model based on the number of species presence points; the more presence points in the dataset, the more features are available to the model and the more complex and fitted the final SDM can become. The default parameters seen in Maxent were derived based on the analysis of a large international dataset of species distributions [2] but have been shown to overfit and select overly complex models [3, 4]. To guard against this risk we tested every possible nested Maxent model for each species to choose the most parsimonious for comparison against null models. Nested models include all possible subset combinations of the predictor variables and internal Maxent functions seen in the full 'default' Maxent model. An example of a nested Maxent model would be one with only one environmental predictor variable and a single Maxent internal function (for example linear features), whilst the full model would select all environmental predictor variables and all Maxent internal functions. Data were split into training (60%) and testing (40%) datasets and the small sample corrected Akaike Information Criterion (AICc) value for each nested model was calculated. The model which returned the lowest AICc value was noted, and the process was repeated for 1000 different splits of each tick presence dataset. The final nested model selected for each species was the one which returned the lowest AICc value of all nested models the most times over the 1000 splits of the species presence data (as advocated by Burnham and Anderson (2002) [5]). AICc values for the nested models were calculated using the ENMTools software v1.3 [6] and the RAW Maxent suitability output format was used for this process.

Supplementary Results: Selection of Maxent models

None of the Maxent models that returned the lowest AICc value during model fitting for each tick species were identified as the most complex model possible (*i.e*. the Maxent default settings). In all cases the model with the lowest AICc value did not use at least two of the Maxent internal fitting options. No tick had a final model containing the threshold and hinge features. The final Maxent models for all species except *Hy. lusitanicum* included the linear, quadratic and product features, whilst the final Maxent model for *Hy. lusitanicum* only selected linear and quadratic features. All three PCA climate variables were used in the final models for all species.

References

1. Elith J, Phillips SJ, Hastie T, Dudik M, Chee YE, Yates CJ. **A statistical explanation of MaxEnt for ecologists**. *Divers Distrib*. 2011; **17**(1): 43-57.
2. Phillips SJ, Dudik M. **Modeling of species distributions with Maxent: new extensions and a comprehensive evaluation**. *Ecography*. 2008; **31**(2): 161-75. doi:10.1111/j.0906-7590.2008.5203.x.
3. Raes N, ter Steege H. **A null-model for significance testing of presence-only species distribution models**. *Ecography*. 2007; **30**(5): 727-36.
4. Warren DL, Seifert SN. **Ecological niche modeling in Maxent: the importance of model complexity and the performance of model selection criteria**. *Ecol Appl*. 2011; **21**(2): 335-42. doi:10.1890/10-1171.1.
5. Burnham KP, Anderson DR. *Model selection and multimodel inference. A practical information-theoretic approach*. 2nd ed. New York, USA: Springer-Verlag; 2002.
6. Warren DL, Glor RE, Turelli M. **ENMTools: a toolbox for comparative studies of environmental niche models**. *Ecography*. 2010; **33**(3): 607-11. doi:10.1111/j.1600-0587.2009.06142.x.
