# Supplementary material for: Climate suitability for European ticks: assessing species distribution models against null models and projection under AR5 climate
Source: Parasit Vectors. 2015 Aug 28;8:440. doi: 10.1186/s13071-015-1046-4 (PMC4551698; doi:10.1186/s13071-015-1046-4)
Supplement: Additional file 2: — Supplementary methods for null data generation and evaluation of observed models against null models. Detailed description of the methods used to generate null data which retained spatial patterns evident in observed data, followed by information on null model construction and testing of observed models against null models. Includes Figure S1: Example of null species distribution data generated for Haemaphysalis punctata. A: observed species distribution; B: example null species distribution; C: semivariogram showing the relationship between semivariance and distance for observed species data (blue line) and 99 null species distributions (grey lines). (PDF 249 kb) [file 13071_2015_1046_MOESM2_ESM.pdf]

Additional file 2: Supplementary methods for null data generation and evaluation of observed models against null models

Detailed description of the methods used to generate null data which retained spatial patterns evident in observed data, followed by information on null model construction and testing of observed models against null models.

Null presence data

For each species, null presence distributions were generated, each consisting of the same number of presence points as that species' observed distribution, placed within the same study area used for the original observations and climate data. Each null distribution was generated by at first randomly placing the desired number of points within the study area, then iteratively moving one presence point at a time, retaining that change only if it reduced the deviation from the spatial pattern in that species' observed presence data. Spatial pattern was assessed using three measures: an empirical semi-variogram, which measures the level of spatial auto-correlation between locations dependent on the distance between them; a density histogram (based on the method of Beale *et al.* (2008) [1]), which measures the probability distribution of the density of presences around each presence point; and the area of the convex hull encompassing all presence points. Deviation from the observed data was calculated for each of these measures as the sum of squares of the difference between the observed and generated measure; the product of all of these measures was taken to give the overall deviation of a generated distribution from the spatial pattern in the observed data. This process was allowed to proceed for a total of 50,000 iterations for each set of null presence data. This number was found to be more than adequate for producing optimised data, with the deviation from the observed spatial pattern reaching a plateau in around half this number of iterations, for most species. The ideal result of this process would be a null presence distribution which does not correlate with the observed distribution, has an empirical semi-variogram that matches that of the observed, and covers a similarly sized area to that of the observed distribution.

We generated around 200 such null presence distributions for each species (more for species where not enough acceptable nulls were found), then selected 99 from these using the following criteria:

- We rejected any null presence distribution which had a correlation coefficient above $1x10^{-4}$ with the observed presence distribution.
- We rejected any null distribution for which the sum of squares deviation for the semi-variogram was greater than $1x10^{-5}$

- From the remaining null presence distributions, we selected those 99 that minimised the total cross-correlation between the resulting set of 99 null distributions.

There is a risk that the spatial geometry of any given study area could constrain the placement of the generated null data. This could manifest itself through map cells in the middle of a study area being selected for inclusion in null presence data at a greater frequency than cells at the edge. This would give rise to the 'mid-domain effect' [2] which increases the probability of erroneously rejecting a true association between climate and the observed species distribution, making the assessment overly conservative [3]. By minimising cross-correlation between generated null datasets, we have minimised the risk of this effect occurring.

The final set of 99 null distributions capture as many qualitatively different distributions as possible, which also adequately conform to the spatial pattern of the observed data. Figure S1 provides an example null species distribution.

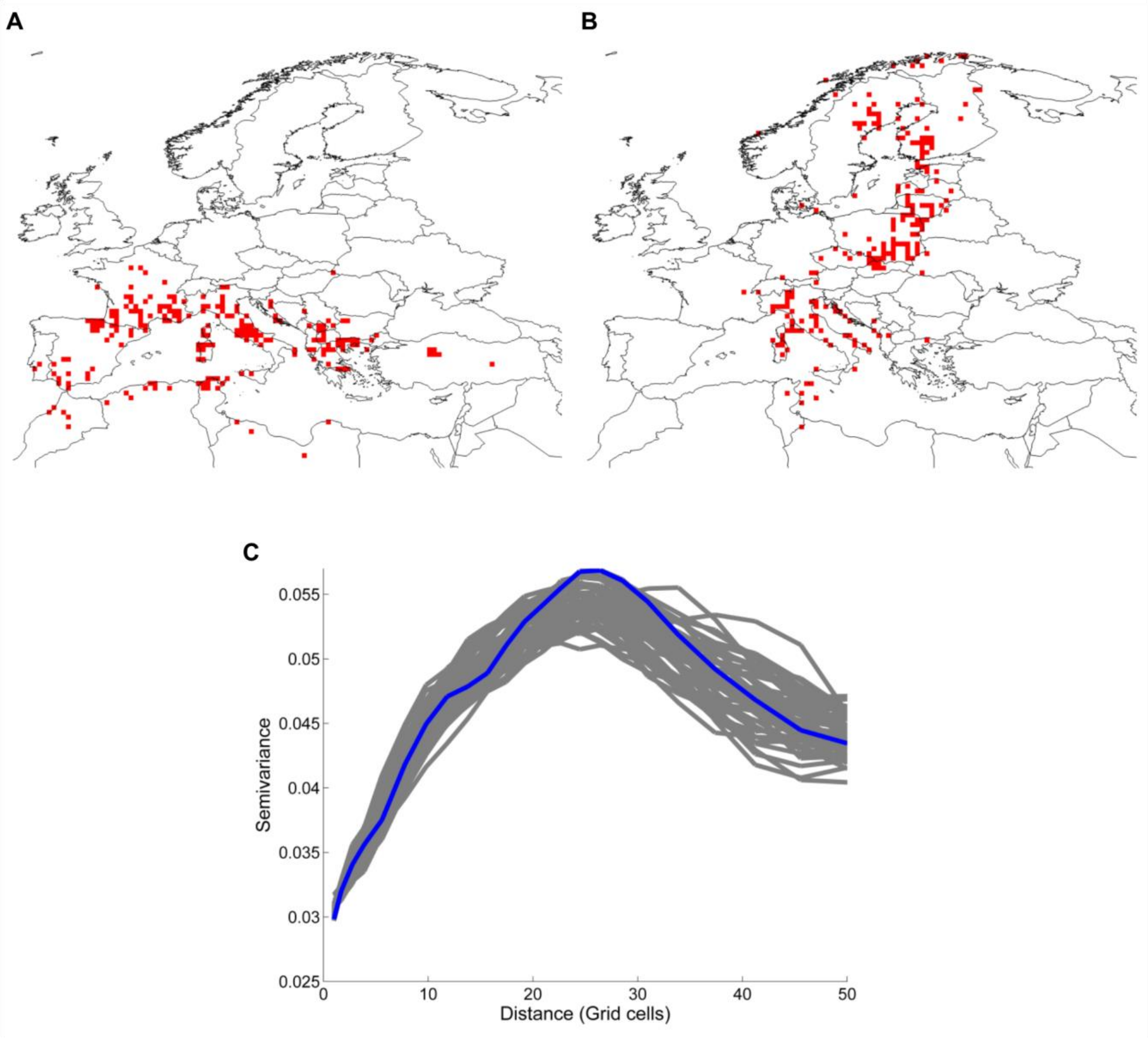

Figure S1: Example of null species distribution data generated for *Haemaphysalis punctata*.

A: observed species distribution; **B**: example null species distribution; **C**: semi-variogram showing the relationship between semivariance and distance for observed species data (blue line) and 99 null species distributions (grey lines).

Null climate data

The spatial pattern in the observed climate data was captured by an empirical semi-variogram, to which we fitted a model semi-variogram (using the R function *variofit* from the R package g*eoR)* From this model semi-variogram, we generated Gaussian Random Fields (using the R function *GaussRF* from the *RandomFields* package), and randomly cut out sections of this matching our study area. A total of 10,000 such cut-outs were generated for each of our three PCA climate layers, filtered such that none of these correlated with the observed climate (i.e.: they had a correlation coefficient of between -0.1 <= c <= 0.1). We then selected the

1,000 with the lowest sum of squares deviation from the observed empirical semi-variogram.

From these null climate layers, we assembled 99 sets of null climate data, each containing a layer for the three PCA climate layers used, with the cross-correlation between each of these layers closely matching that found in the observed climate (i.e.: the correlation coefficient between two null climate layers deviated by less than 0.05 from the correlation coefficient for the observed climate layers).

The ideal result of this process is a set of three null climate layers (corresponding to the three observed PCA layers), each with an empirical semi-variogram matching that of the corresponding observed climate layer, not correlated with that observed layer, and correlating with the other layers to the same degree as in the observed climate. For example, if areas of warm climate coincided with areas of high rainfall in the observed data, the same connection should be preserved in the null climate data.

Data splitting

We generated 1000 training/testing splits from each species presence dataset (both the observed presence distribution and the 99 null presence distributions), with 60% of presence points used for building the SDM, and the remaining 40%, along with an equal number pseudo-absences selected randomly from elsewhere in the study area, used to assess that SDM and calculate an AUC.

Null model generation

For our purposes, a null model is the result of applying an SDM to either null presence data and observed climate data; or observed presence data and null climate data. We are interested only in the AUC scores of these models, and not the resulting suitability map. For each species we created four sets of 99 null models:

- a Maxent model of that species' 99 null presence distributions on the observed climate
- an MD model of that species' 99 null presence distributions on the observed climate
- a Maxent model of that species' real distribution on each of the 99 null climate sets
- an MD model of that species' real distribution on each of the 99 null climate sets

We also created two observed models: a Maxent and an MD model of that species' observed distribution on the observed climate.

Significance of the observed model

Each set of 99 null models (four sets for each species, as above), was combined with its counterpart observed model (MD or Maxent, for each species), into a population of 100 models. A Kruskal-Wallis test was used to detect whether the median AUC (calculated from the 1000 training/testing splits) of any of these 100

models was significantly different to any other. If a significant difference was detected, we used pairwise t-tests with Bonferonni correction (using the *multcompare* function in MATLAB, version 2014a), to count how many of the null models had significantly lower median AUCs than the observed model. We considered the association between climate and distribution identified by an SDM to be significant if the AUC of the observed model was significantly greater than that of 95 of the null models, placing the observed model in the top 5% of all models. This corresponds to a one-tailed significance level of $p <= 0.05$.

1. Beale CM, Lennon JJ, Gimona A. **Opening the climate envelope reveals no macroscale associations with climate in European birds**. *P Natl Acad Sci USA*. 2008;**105**(39):14908-12.
2. Colwell RK, Rahbek C, Gotelli NJ. **The mid-domain effect and species richness patterns: What have we learned so far?** *Am Nat*. 2004;**163**(3):E1-E23.
3. Peterson AT, Barve N, Bini LM, Diniz-Filho JA, Jiménez-Valverde A, Lira-Noriega A et al. **The climate envelope may not be empty**. *P Natl Acad Sci USA*. 2009;**106**(16):E47. doi:10.1073/pnas.0809722106.
