# Supplementary material for: Climate suitability for European ticks: assessing species distribution models against null models and projection under AR5 climate
Source: Parasit Vectors. 2015 Aug 28;8:440. doi: 10.1186/s13071-015-1046-4 (PMC4551698; doi:10.1186/s13071-015-1046-4)
Supplement: Additional file 3: — PCA results. Table S1: Loadings of Principal Component Analysis of western Palearctic climate. 20 climate variables averaged over 40 years (1971–2010); data obtained from Climate Research Unit time series (version 3.22). Figure S2: Mapped Principal Component (PC) scores in western Palearctic. Results from PCA of 40-year averages (1971–2010) of 20 observed climate variables. A: PC1; B: PC2; C: PC3. (PDF 248 kb) [file 13071_2015_1046_MOESM3_ESM.pdf]

Additional file 3: PCA results

Table S1: Loadings of Principal Component Analysis of western Palearctic climate.

20 climate Variables averaged over 40 years (1971-2010); data obtained from Climate Research Unit time series (version 3.22), and calculated following Busby (1991) [1].

Climate variable	PCA 1	PCA 2	PCA 3
Saturation deficit during spring and summer (Hectopascal)	0.25670	-0.01349	0.27110
Annual Mean Temperature (°C)	0.25543	-0.20044	0.00862
Mean Diurnal Temperature Range (°C)	0.24456	0.07622	0.31761
Isothermality (°C)	0.20736	-0.24554	-0.01106
Temperature Seasonality (°C)	0.00052	0.41558	0.32995
Maximum Temperature of the Warmest Month (°C)	0.27472	-0.04186	0.18477
Minimum Temperature of the Coldest Month (°C)	0.19048	-0.33282	-0.12667
Temperature Annual Range (°C)	0.08212	0.38700	0.39028
Mean Temperature Wettest Quarter (°C)	0.19015	0.01532	-0.36685
Mean Temperature Driest Quarter (°C)	0.23720	-0.22230	0.18613
Mean Temperature Warmest Quarter (°C)	0.27292	-0.08632	0.12297
Mean Temperature Coldest Quarter (°C)	0.22111	-0.28915	-0.07727
Annual Precipitation (mm)	-0.24141	-0.21818	0.20280
Precipitation Wettest Month (mm)	-0.20662	-0.25992	0.28062
Precipitation Driest Month (mm)	-0.25232	-0.11426	0.04351
Precipitation Seasonality (Coefficient of Variation)	0.24904	-0.05598	0.13360
Precipitation Wettest Quarter (mm)	-0.21452	-0.25512	0.27252
Precipitation Driest Quarter (mm)	-0.25181	-0.14376	0.05229
Precipitation of the Warmest Quarter (mm)	-0.25886	-0.00469	-0.05423
Precipitation of the Coldest Quarter (mm)	-0.16933	-0.31734	0.32277

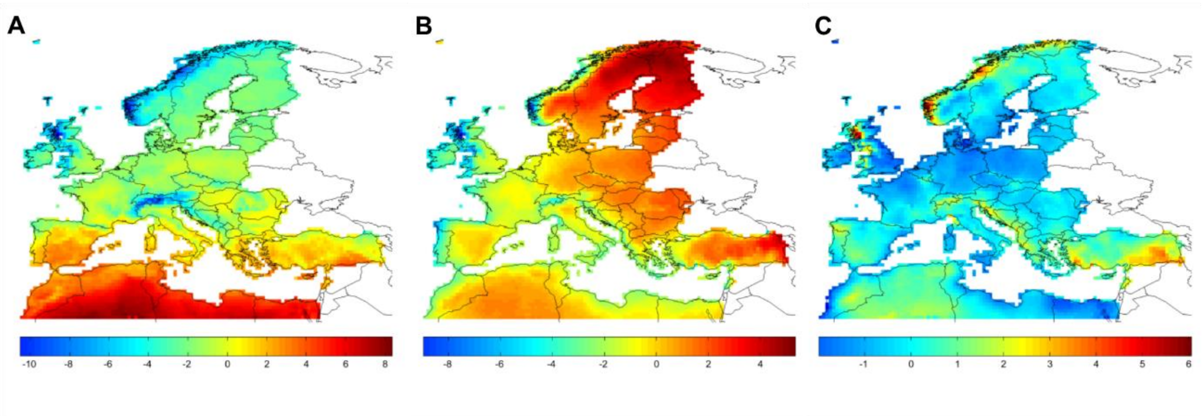

Figure S2: Mapped Principal Component (PC) scores in western Palearctic. Results from PCA of 40-year averages (1971-2010) of 20 observed climate variables listed in Table S1. **A**: PC1; **B**: PC2; **C**: PC3.

1. Busby JR. **BIOCLIM—a bioclimatic analysis and prediction tool**. *Plant Prot Q*. 1991;**6**:8-9.
