# Supplementary material for: Climate suitability for European ticks: assessing species distribution models against null models and projection under AR5 climate
Source: Parasit Vectors. 2015 Aug 28;8:440. doi: 10.1186/s13071-015-1046-4 (PMC4551698; doi:10.1186/s13071-015-1046-4)
Supplement: Additional file 4: — Projected current and future climate suitability under RCP 2.6, 6.0 & 8.5. Figures S3-S8 & Table S2. Figure S3. Current and future (RCP 2.6) projected climate suitability for tick species in the western Palearctic. Each row corresponds to a tick species: A: Ixodes ricinus; B: Rhipicephalus annulatus; C: Dermacentor marginatus; D: Haemaphysalis punctata. Columns correspond to 40-year temporal averages up to and including: 1: 2010; 2: 2050; 3: 2098. Figures in column 1 represent the average suitability derived from Maxent and MD SDMs based on observed climate; columns 2 and 3 contain suitability averaged across Maxent and MD SDMs produced from four GCMs following RCP 2.6. Values range from 0 (unsuitable) to 1 (highly suitable). Figure S4. Current and future (RCP 2.6) projected climate suitability for tick species in the western Palearctic. Each row corresponds to a tick species: E: Haemaphysalis sulcata; F: Hyalomma marginatum; G: Rhipicephalus bursa. Columns correspond to 40-year temporal averages up to and including: 1: 2010; 2: 2050; 3: 2098. Figures in column 1 represent the average suitability derived from Maxent and MD SDMs based on observed climate; columns 2 and 3 contain suitability averaged across Maxent and MD SDMs produced from four GCMs following RCP 2.6. Values range from 0 (unsuitable) to 1 (highly suitable). Figure S5. Current and future (RCP 6.0) projected climate suitability for tick species in the western Palearctic. Each row corresponds to a tick species: A: Ixodes ricinus; B: Rhipicephalus annulatus; C: Dermacentor marginatus; D: Haemaphysalis punctata. Columns correspond to 40-year temporal averages up to and including: 1: 2010; 2: 2050; 3: 2098. Figures in column 1 represent the average suitability derived from Maxent and MD SDMs based on observed climate; columns 2 and 3 contain suitability averaged across Maxent and MD SDMs produced from four GCMs following RCP 6.0. Values range from 0 (unsuitable) to 1 (highly suitable). Figure S6. Current an [file 13071_2015_1046_MOESM4_ESM.pdf]

Additional file 4: Projected current and future climate suitability under RCP 2.6, 6.0 & 8.5. Figs S3-S8 & Table S2

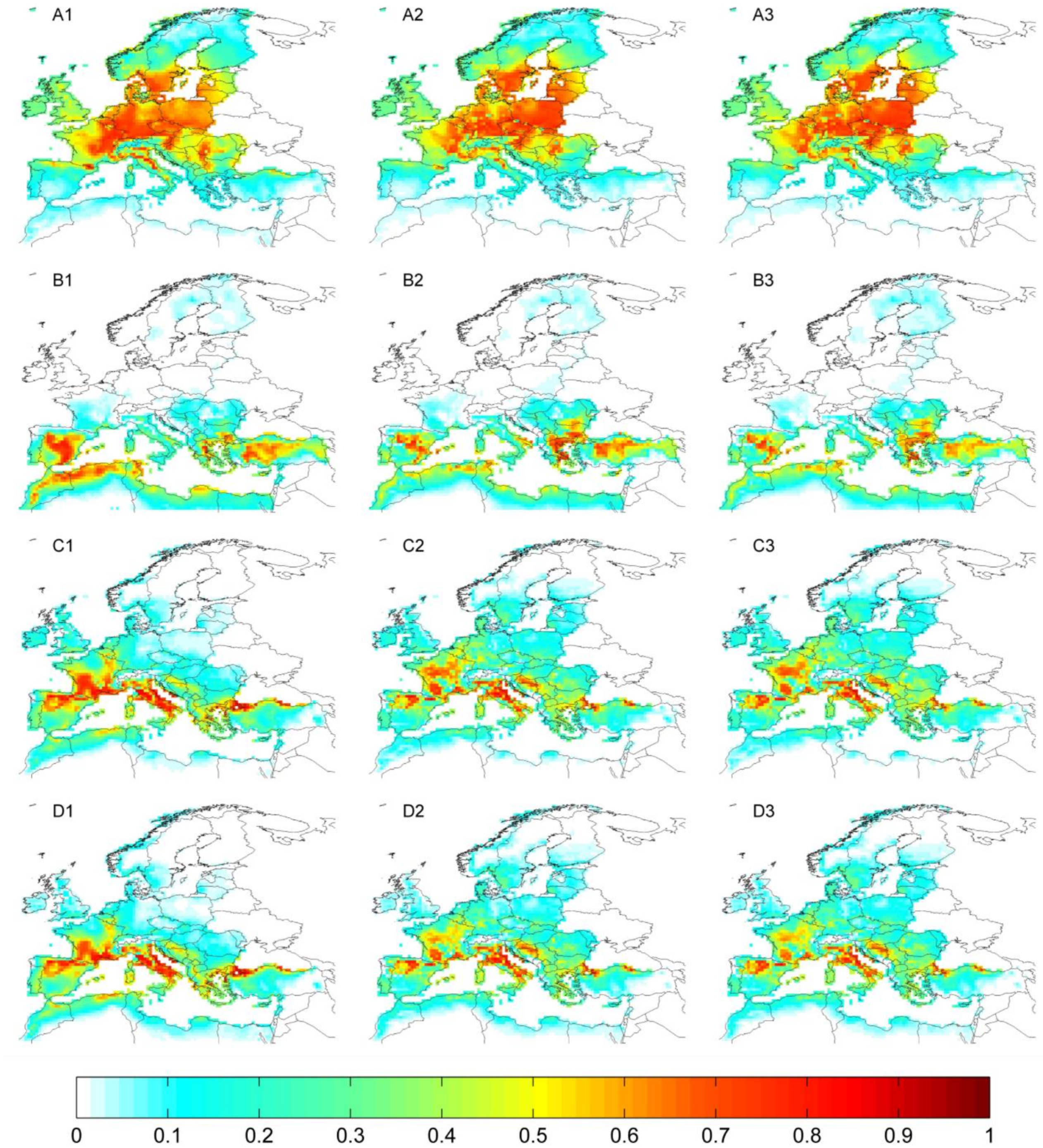

Figure S3. Current and future (RCP 2.6) projected climate suitability for tick species in the western Palearctic. Each row corresponds to a tick species: **A**: *Ixodes ricinus;* **B**: *Rhipicephalus annulatus*; **C**: *Dermacentor marginatus*; **D**: *Haemaphysalis punctata.* Columns correspond to 40-year temporal averages up to and including: **1**: 2010; **2**: 2050; **3**: 2098. Figures in column 1 represent the average suitability derived from Maxent and MD SDMs based on observed climate; columns 2 and 3 contain suitability averaged across Maxent and MD SDMs produced from four GCMs following RCP 2.6.

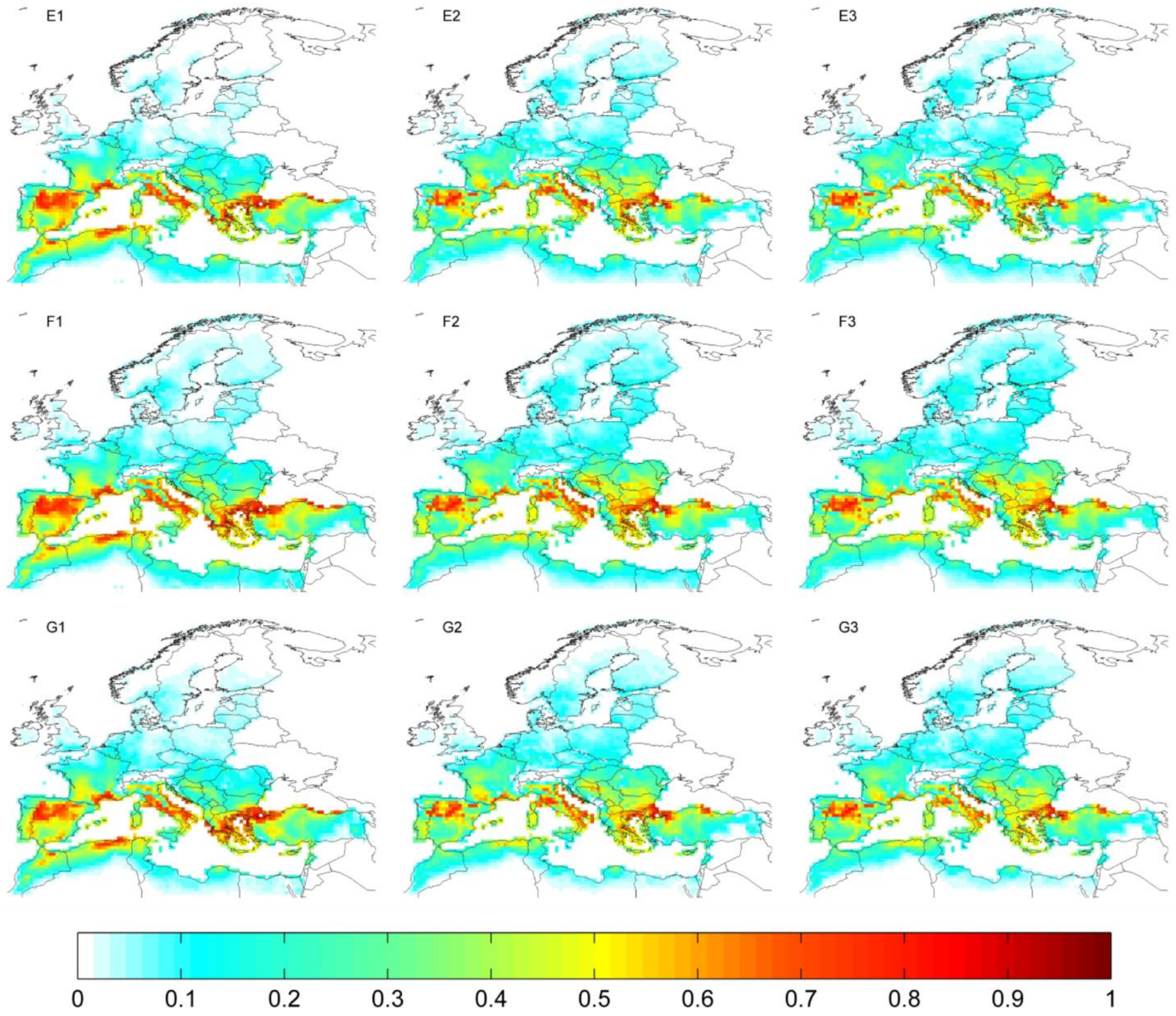

Figure S4. Current and future (RCP 2.6) projected climate suitability for tick species in the western Palearctic. Each row corresponds to a tick species: **E**: *Haemaphysalis sulcata*; **F**: *Hyalomma marginatum*; **G**: *Rhipicephalus bursa.* Columns correspond to 40-year temporal averages up to and including: **1**: 2010; **2**: 2050; **3**: 2098. Figures in column 1 represent the average suitability derived from Maxent and MD SDMs based on observed climate; columns 2 and 3 contain suitability averaged across Maxent and MD SDMs produced from four GCMs following RCP 2.6.

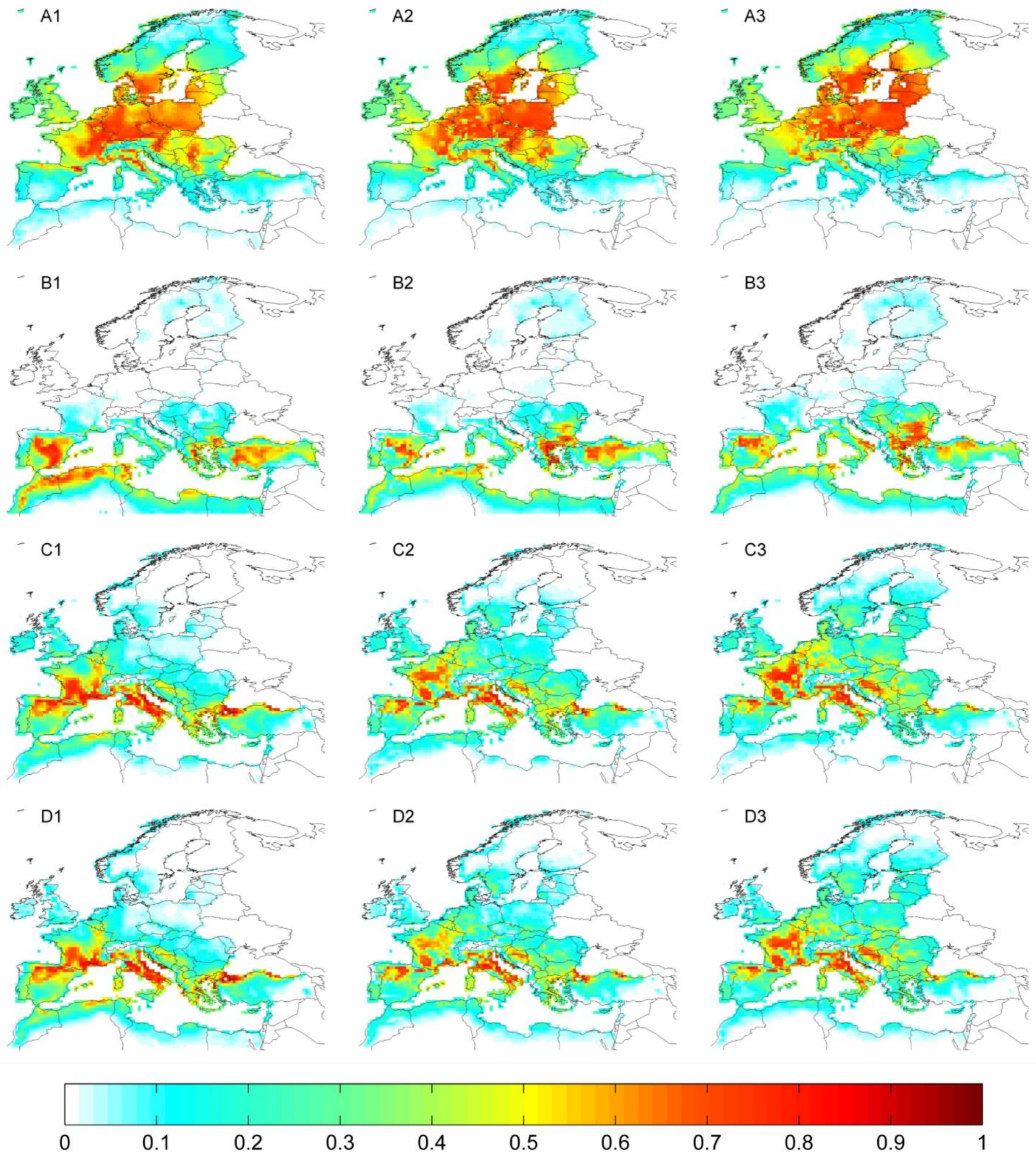

Figure S5. Current and future (RCP 6.0) projected climate suitability for tick species in the western Palearctic. Each row corresponds to a tick species: **A**: *Ixodes ricinus;* **B**: *Rhipicephalus annulatus*; **C**: *Dermacentor marginatus*; **D**: *Haemaphysalis punctata*. Columns correspond to 40-year temporal averages up to and including: **1**: 2010; **2**: 2050; **3**: 2098. Figures in column 1 represent the average suitability derived from Maxent and MD SDMs based on observed climate; columns 2 and 3 contain suitability averaged across Maxent and MD SDMs produced from four GCMs following RCP 6.0.

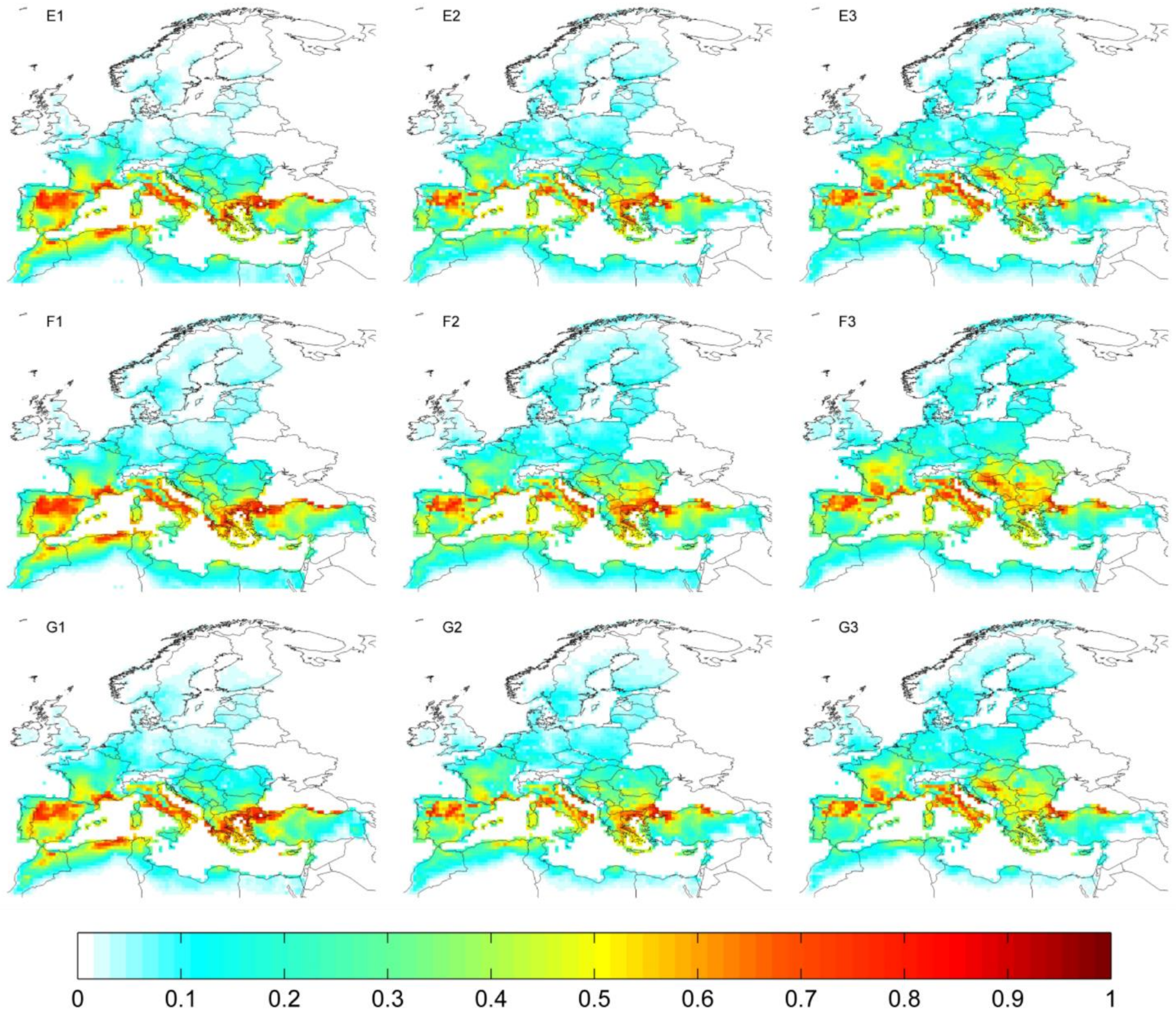

Figure S6. Current and future (RCP 6.0) projected climate suitability for tick species in the western Palearctic. Each row corresponds to a tick species: **E**: *Haemaphysalis sulcata*; **F**: *Hyalomma marginatum*; **G**: *Rhipicephalus bursa.* Columns correspond to 40-year temporal averages up to and including: **1**: 2010; **2**: 2050; **3**: 2098. Figures in column 1 represent the average suitability derived from Maxent and MD SDMs based on observed climate; columns 2 and 3 contain suitability averaged across Maxent and MD SDMs produced from four GCMs following RCP 6.0.

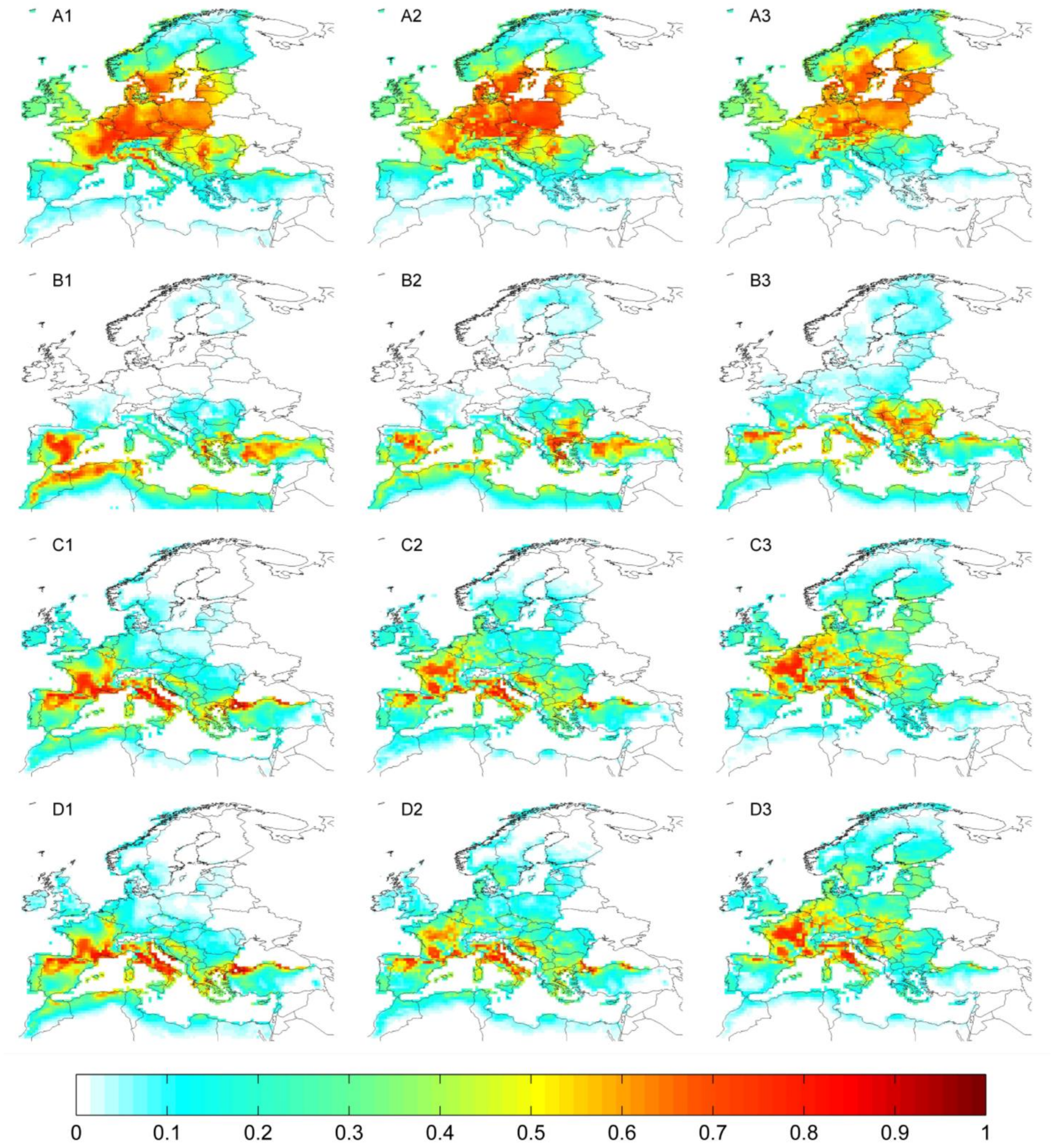

Figure S7. Current and future (RCP 8.5) projected climate suitability for tick species in the western Palearctic. Each row corresponds to a tick species: **A**: *Ixodes ricinus;* **B**: *Rhipicephalus annulatus*; **C**: *Dermacentor marginatus*; **D**: *Haemaphysalis punctata.* Columns correspond to 40-year temporal averages up to and including: **1**: 2010; **2**: 2050; **3**: 2098. Figures in column 1 represent the average suitability derived from Maxent and MD SDMs based on observed climate; columns 2 and 3 contain suitability averaged across Maxent and MD SDMs produced from four GCMs following RCP 8.5.

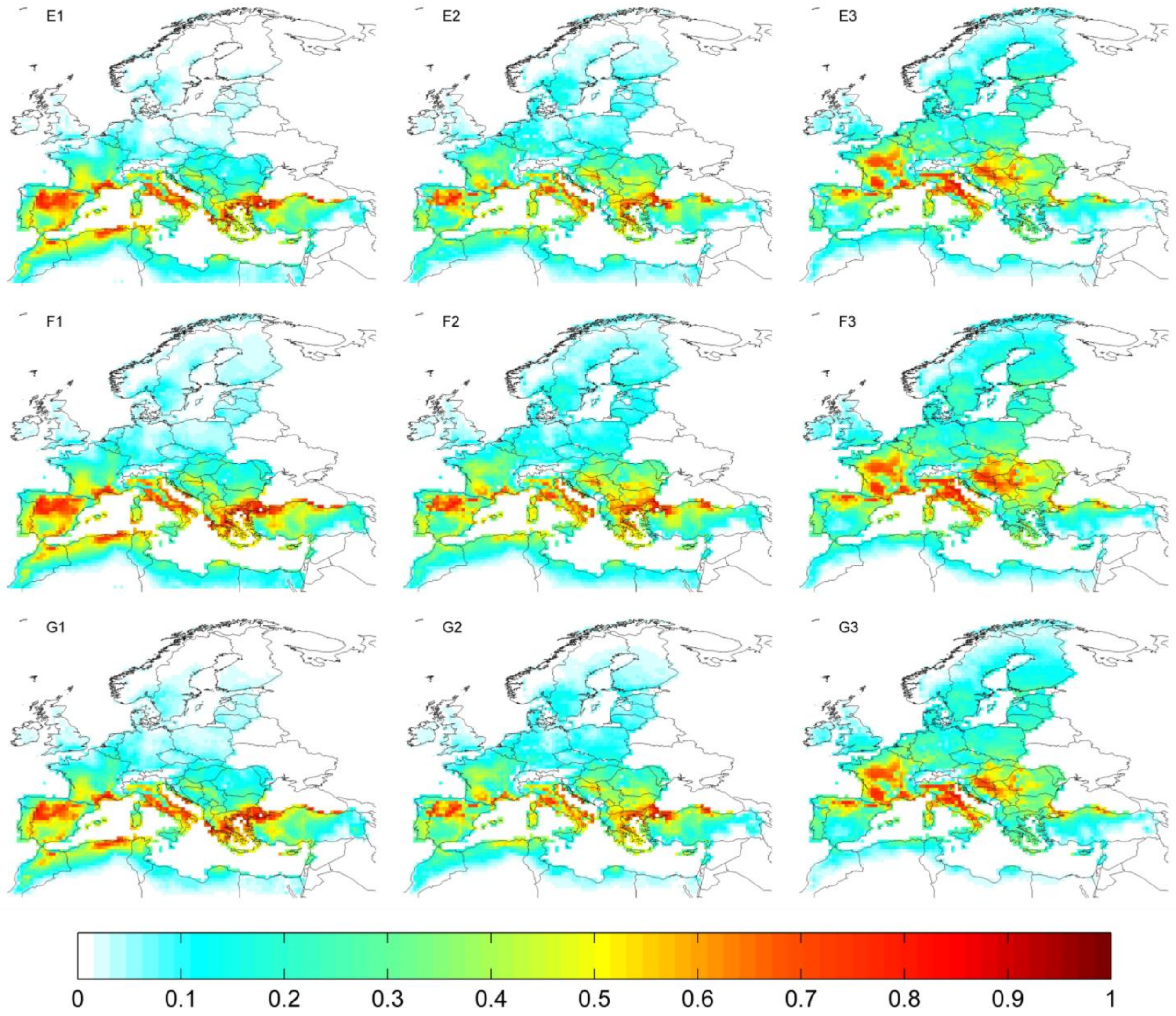

Figure S8. Current and future (RCP 8.5) projected climate suitability for tick species in the western Palearctic. Each row corresponds to a tick species: **E**: *Haemaphysalis sulcata*; **F**: *Hyalomma marginatum*; **G**: *Rhipicephalus bursa.* Columns correspond to 40-year temporal averages up to and including: **1**: 2010; **2**: 2050; **3**: 2098. Figures in column 1 represent the average suitability derived from Maxent and MD SDMs based on observed climate; columns 2 and 3 contain suitability averaged across Maxent and MD SDMs produced from four GCMs following RCP 8.5.

Table S2: Similarity between observed and future projected climate suitability for seven tick species. Schoener's *D* statistic represents the degree of overlap between climate suitability maps, ranging from 0 (no overlap) to 1 (complete overlap) [1, 2]. Smallest values therefore indicate least overlap and so the greatest change between current and future projections of climate suitability. Average climate suitability produced by Maxent and MD SDMs for the 40-year period up to and including 2010 has been compared with climate suitability averaged across both SDMs over future 40-year periods up to and including 2050 and 2098 under all four RCP climates. This analysis was undertaken using ENMTools software v1.3 [3].

	**RCP 2.6**		**RCP 4.5**		**RCP 6.0**		**RCP 8.5**	
**Tick species**	**2050**	**2098**	**2050**	**2098**	**2050**	**2098**	**2050**	**2098**
Rhipicephalus annulatus	0.82321	0.81683	0.82218	0.74316	0.82694	0.73902	0.81456	0.59897
Dermacentor marginatus	0.77561	0.75158	0.78037	0.69198	0.78397	0.66970	0.76210	0.56007
Haemaphysalis punctata	0.77749	0.75397	0.78054	0.68909	0.78163	0.66836	0.76144	0.54843
Haemaphysalis sulcata	0.80977	0.79599	0.80921	0.72630	0.81045	0.71170	0.79545	0.57954
Hyalomma marginatum	0.84498	0.83095	0.84240	0.75984	0.84662	0.74530	0.82705	0.61523
Ixodes ricinus	0.91312	0.91130	0.91444	0.86578	0.92281	0.85670	0.91259	0.78594
Rhipicephalus bursa	0.83674	0.82339	0.83539	0.75124	0.84082	0.73076	0.81982	0.59557

References

1. Schoener TW. **The Anolis lizards of Bimini: resource partitioning in a complex fauna**. *Ecology*. 1968; **49**(4): 704-26.
2. Warren DL, Glor RE, Turelli M. **Environmental niche equivalency versus conservatism: quantitative approaches to niche evolution**. *Evolution*. 2008; **62**(11): 2868-83.
3. Warren DL, Glor RE, Turelli M. **ENMTools: a toolbox for comparative studies of environmental niche models**. *Ecography*. 2010; **33**(3): 607-11. doi:10.1111/j.1600-0587.2009.06142.x.
